# Wilson Disease Protein ATP7B Utilizes Lysosomal Exocytosis to Maintain Copper Homeostasis

**DOI:** 10.1016/j.devcel.2014.04.033

**Published:** 2014-06-23

**Authors:** Elena V. Polishchuk, Mafalda Concilli, Simona Iacobacci, Giancarlo Chesi, Nunzia Pastore, Pasquale Piccolo, Simona Paladino, Daniela Baldantoni, Sven C.D. van IJzendoorn, Jefferson Chan, Christopher J. Chang, Angela Amoresano, Francesca Pane, Piero Pucci, Antonietta Tarallo, Giancarlo Parenti, Nicola Brunetti-Pierri, Carmine Settembre, Andrea Ballabio, Roman S. Polishchuk

**Affiliations:** 1 Telethon Institute of Genetics and Medicine (TIGEM), Naples 80131, Italy; 2 Jan and Dan Duncan Neurological Research Institute, Houston, TX 77030, USA; 3 Department of Molecular Medicine and Medical Biotechnology, Federico II University, Naples 80125, Italy; 4 University of Salerno, Fisciano (SA) 84084, Italy; 5 Department of Cell Biology, University of Groningen, University Medical Center Groningen, Groningen 9713, the Netherlands; 6 Department of Chemistry and Molecular and Cell Biology and Howard Hughes Medical Institute, University of California, Berkeley, Berkeley, CA 94720, USA; 7 Department of Chemical Sciences, University of Naples Federico II, Napoli 80126, Italy; 8 Medical Genetics, Department of Translational and Medical Sciences, Federico II University, Naples 80125, Italy; 9 Department of Molecular and Human Genetics, Baylor College of Medicine, Houston, TX 77030, USA; 10 Dulbecco Telethon Institute, TIGEM, Naples 80131, Italy

## Abstract

Copper is an essential yet toxic metal and its overload causes Wilson disease, a disorder due to mutations in copper transporter ATP7B. To remove excess copper into the bile, ATP7B traffics toward canalicular area of hepatocytes. However, the trafficking mechanisms of ATP7B remain elusive. Here, we show that, in response to elevated copper, ATP7B moves from the Golgi to lysosomes and imports metal into their lumen. ATP7B enables lysosomes to undergo exocytosis through the interaction with p62 subunit of dynactin that allows lysosome translocation toward the canalicular pole of hepatocytes. Activation of lysosomal exocytosis stimulates copper clearance from the hepatocytes and rescues the most frequent Wilson-disease-causing ATP7B mutant to the appropriate functional site. Our findings indicate that lysosomes serve as an important intermediate in ATP7B trafficking, whereas lysosomal exocytosis operates as an integral process in copper excretion and hence can be targeted for therapeutic approaches to combat Wilson disease.

## Highlights

• ATP7B moves from the Golgi to lysosomes in response to elevated copper levels
• ATP7B promotes storage of copper in lysosomal lumen
• By interacting with p62/dynactin, ATP7B promotes polarized exocytosis of lysosomes
• Lysosomal exocytosis allows hepatocytes to release excess copper into the bile

## Graphical Abstract

**Figure:**
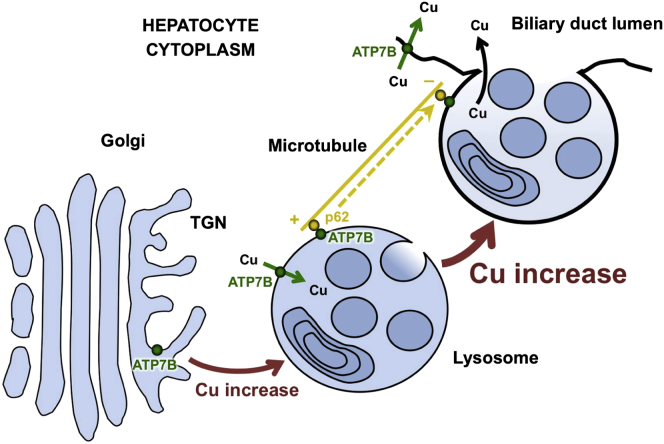

## Teaser

Mutations in the copper transporter ATP7B cause copper overload and toxicity in Wilson disease. Polishchuk et al. show that copper overload induces ATP7B transfer from the Golgi to lysosomes, where ATP7B sequesters excess metal in the lumen and, via interaction with dynactin, promotes copper exocytosis from hepatocytes into bile.

## Introduction

**Figure 1:**
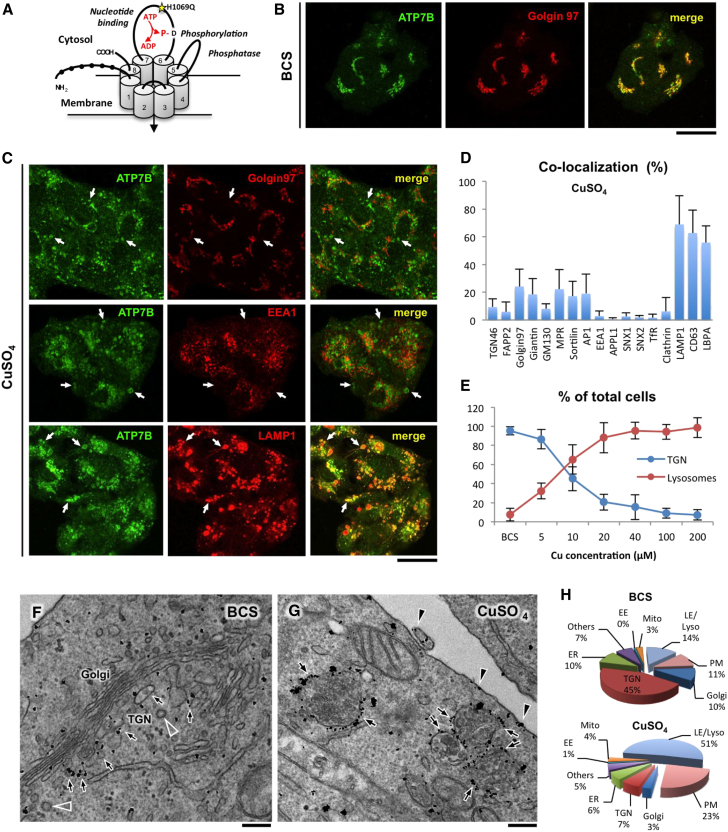
Increasing Cu Concentration Triggers ATP7B Trafficking from the TGN to LE/Lysosome Compartments (A) Schematic structure of ATP7B. Black balls show N-terminal metal-binding domains. Numbers indicate transmembrane helices. The domains, which regulate ATPase activity, are indicated in italic with D residue for catalytic phosphorylation and with most frequent WD-causing mutation, H1069Q (yellow star). (B) HepG2 cells were fixed after overnight exposure to 200 μM BCS and stained for endogenous ATP7B and golgin 97. (C) BCS-treated cells were washed and incubated with 200 μM CuSO_4_ for 2 hr. Confocal microscopy reveals endogenous ATP7B in vacuolar structures (arrows), which did not contain Golgin 97 or EEA1 but were decorated by LAMP1. (D) Quantification shows ATP7B colocalization (mean ± SD; n = 50 cells) with lysosomal markers. (E) Percentage (mean ± SD; n = 20 fields) of the cells with ATP7B in the TGN or in the lysosomes was calculated for treatments with BCS or with different concentration of CuSO_4_ (as indicated along x axis). (F and G) HepG2 cells were infected with adenovirus carrying ATP7B-GFP (adeno-ATP7B-GFP) and incubated with BCS. Then, the cells were fixed either directly (F) or after 2 hr incubation with CuSO_4_ (G) and immunogold labeled to reveal ATP7B-GFP. Arrows indicate ATP7B signal over the TGN membranes in low Cu (F) or over the MVB/lysosome-like structures (G) in elevated Cu. Arrowheads show ATP7B at the cell surface in cells exposed to CuSO_4_ (G). (H) Pie plots exhibit percentage of ATP7B-associated gold particles in different compartments in cells treated with BCS or CuSO_4_. EE, early endosome. The scale bars represent 5 μm (B and C) or 250 nm (F and G).

Copper is an indispensable micronutrient because a number of enzymes require it as a cofactor for fundamental metabolic processes such as respiration; free radical scavenging; pigmentation; and synthesis of collagen, elastin, and neurotransmitters (Lutsenko, 2010; Nevitt et al., 2012). However, due to its redox potential, copper can induce cellular toxicity. To avoid toxic accumulation of Cu, vertebrates developed a fine-tuned mechanism that allows excess Cu to be removed from the organism through the Cu-transporting ATPase ATP7B. ATP7B is a large multidomain protein with eight transmembrane helices, which form a channel that pumps Cu from the cytosol at the expense of ATP hydrolysis (Figure 1A). ATP7B is highly expressed in liver, where it normally resides in the trans-Golgi network (TGN) of hepatocytes and loads Cu on newly synthetized ceruloplasmin, the major Cu-carrying protein in the blood (Lutsenko, 2010). When intracellular Cu levels increase, ATP7B is thought to traffic toward the biliary surface of hepatocytes and associated “vesicles” involved in the excretion of Cu into bile. Mutations in the *ATP7B* gene frequently result in the failure of its protein product to traffic to the sites of Cu excretion. This defect causes toxic accumulation of Cu in the liver and, as a consequence, development of Wilson disease that is fatal if not treated in time (Gupta and Lutsenko, 2009).

Despite the fundamental role of ATP7B trafficking in Cu homeostasis, the intracellular itinerary of ATP7B transport remains poorly understood and controversial (La Fontaine and Mercer, 2007; Polishchuk and Lutsenko, 2013). First, in contrast to common view, several studies conducted in hepatic cells indicate that Cu does not alter the intracellular distribution of ATP7B (Harada et al., 2000, 2005). Second, the uncertainty in ATP7B trafficking concerns the identity of the peripheral vesicular structures, whose ability to receive ATP7B upon Cu overload was associated with a Cu excretion process (La Fontaine and Mercer, 2007; Polishchuk and Lutsenko, 2013). The majority of studies failed to demonstrate any significant overlap between ATP7B vesicles and common exo- or endocytic markers (Guo et al., 2005; La Fontaine et al., 2001), whereas few publications reported a fluorescent ATP7B fusion protein within the late endosome compartment (Harada et al., 2000, 2005). Therefore, the simple term “vesicles” is often applied to ATP7B-positive structures because lack of coherent data identifying their molecular composition and ultrastructure makes it problematic to classify them as specific exo- or endocytic organelles. Finally, the question on whether or not ATP7B really reaches the canalicular surface of hepatocytes became the issue of ongoing debate (Hubbard and Braiterman, 2008).

As a consequence of above gaps in understanding of ATP7B trafficking, it is yet to be determined (1) which transport route is employed by ATP7B to reach “vesicles” and from where it emerges, (2) whether and how ATP7B gets delivered from “vesicles” to the canalicular surface of hepatocytes, and (3) how ATP7B trafficking is coordinated with Cu excretion from the cell.

Here, we show that an increase in Cu concentration induces direct ATP7B trafficking from the TGN to a subset of lysosomes, where ATP7B imports Cu for storage in the lysosome lumen and through the interaction with p62 subunit of dynactin complex enables lysosomes for polarized exocytosis at the canalicular surface of hepatocytes. Activation of lysosomal exocytosis stimulates both the delivery of ATP7B and its Wilson-disease-causing mutant to the canalicular membrane domains of hepatocytes and the release of excess Cu into the bile. Thus, our findings indicate ATP7B-containing lysosomes and lysosomal exocytosis as key components of Cu homeostasis.

## Results

### Cu Induces ATP7B Redistribution from the TGN to Late-Endosome/Lysosome Compartments

We first investigated trafficking and localization of ATP7B in hepatoma HepG2 cells under different conditions varying in Cu levels. HepG2 cells express endogenous ATP7B and maintain key properties of normal hepatocytes, representing a reliable system to investigate trafficking of human ATP7B (Cater et al., 2006; Roelofsen et al., 2000). Figure 1B shows that Cu chelation with bathocuproine disulphonate (BCS) resulted in ATP7B accumulation in the Golgi region, where ATP7B colocalized with the TGN marker golgin-97. To stimulate ATP7B trafficking from the TGN, BCS-treated cells were washed and exposed to 200 μM CuSO_4_ for 2 hr. This resulted in complete loss of the ATP7B from the TGN and its relocation to peripheral vesicular structures (Figure 1C, arrows). To determine whether these structures belong to an annotated exo- or endocytic compartment, we tested a battery of markers for overlap with the endogenous ATP7B. Confocal microscopy revealed significant colocalization between ATP7B and the late-endosome (LE)/lysosome markers LAMP1, CD63, and LBPA in the vesicular structures (Figures 1C and 1D and Figure S1A available online). In addition, we analyzed the distribution of the S340A mutant of ATP7B, which constantly resides in “vesicular” compartments (Hasan et al., 2012), and found its robust overlap with LAMP1 (Figure S1B). These observations suggest that ATP7B traffics from the TGN to the LE/lysosome compartment in response to elevated Cu. This process was extremely sensitive to Cu. Even relatively low (5–20 μM) Cu concentration induced ATP7B trafficking to LE/lysosomes (Figure 1E). Importantly, we also found LAMP1-, CD63-, and LBPA-positive organelles without ATP7B signal, indicating that only a subset (about 40%) of the LE/lysosomes received ATP7B from the TGN (Figure S1C).

**Figure 2:**
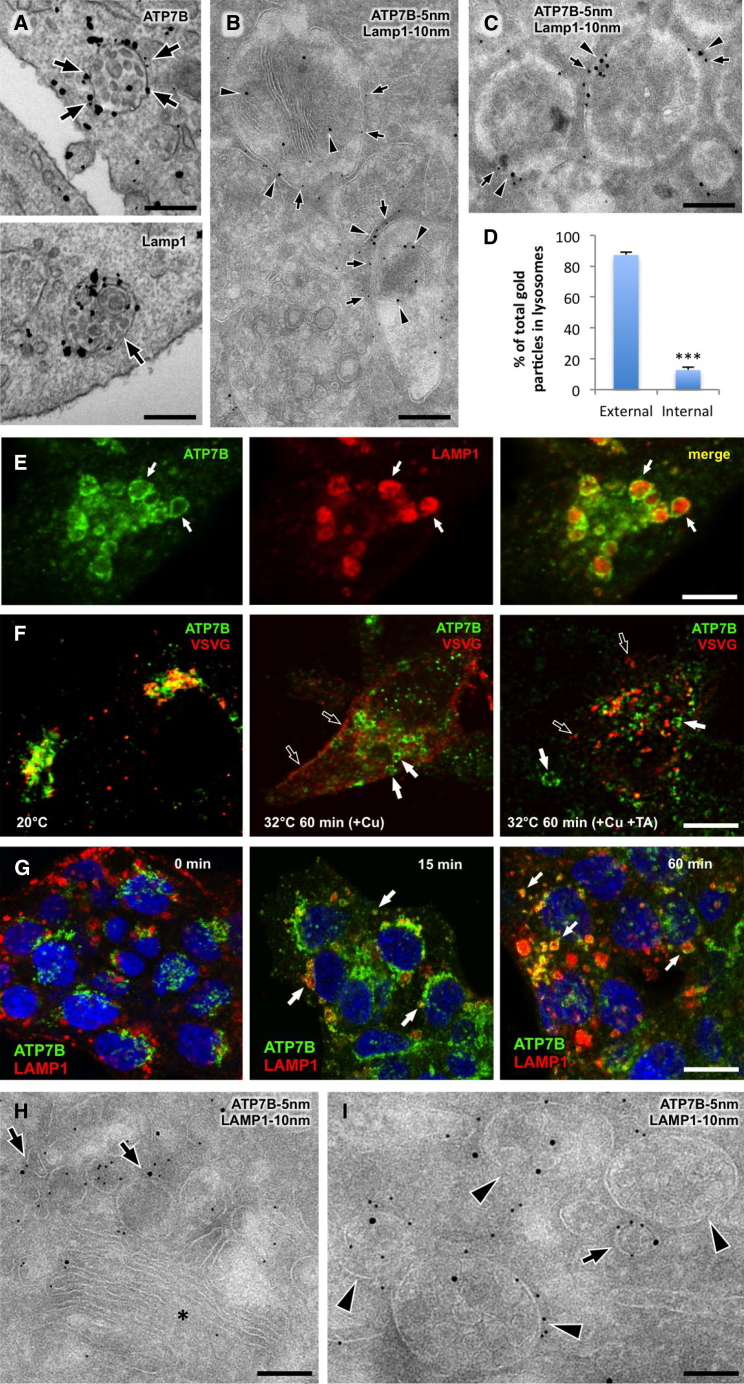
Lysosomes Retain ATP7B at Their Limiting Membranes and Receive ATP7B from the TGN through a Direct Route (A) CuSO_4_-treated HepG2 cells were immunogold labeled to reveal either ATP7B-GFP or LAMP1. Arrows in top panel indicate ATP7B distribution along limiting membrane of the MVBs, whereas some LAMP1 labeling can be seen at the internal membranes of lysosomes (arrow in bottom panel). (B) CuSO_4_-treated HepG2 cells were processed for cryo-immuno-EM. Arrows and arrowheads indicate ATP7B-GFP and LAMP1, respectively, within the same lysosome-like structures. (C) Liver tissue from mice, which was injected with adeno-ATP7B-GFP and treated with CuSO_4_, was labeled for ATP7B-GFP and LAMP1. ATP7B-GFP (arrows) and LAMP1 (arrowheads) were detected together within lysosome-like structures. (D) Quantification of the percentage of gold particles in lysosomes (mean ± SD; n = 100 structures) shows most of ATP7B to reside at the external membrane. (E) CuSO_4_-treated HepG2 cells exhibit endogenous ATP7B as circles (arrows) at the surface of LAMP1-positive structures. (F) HepG2 cells were infected with VSV (see Experimental Procedures) and fixed directly after 20°C block (left panel) or incubated at 32°C with CuSO_4_ for 60 min with (right panel) or without (midpanel) tannic acid (TA). Empty arrows indicate VSVG at the cell surface (midpanel) and post-Golgi carriers (right panel), whereas filled arrows indicate lysosome-like ATP7B structures. (G) HepG2 cells were fixed directly after incubation with BCS or exposed to CuSO_4_ for either 15 min or 60 min and stained for endogenous ATP7B and LAMP1. Arrows indicate ATP7B/LAMP1-positive structures. (H and I) HepG2 cells expressing ATP7B-GFP were incubated with BCS and fixed directly (H) or 15 min after incubation with CuSO_4_ (I) and labeled for ATP7B-GFP and LAMP1. ATP7B and LAMP1 were detected in some TGN domains (H, arrows) of the Golgi stack (H, asterisk). Arrow in (I) indicates ATP7B/LAMP1 post-Golgi carrier near the ATP7B/LAMP1-positive MVBs (arrowheads). The scale bars represent 250 nm (A), 150 nm (B, C, H, and I), 3.5 μm (E and F), and 7 μm (G).

To further verify LE/lysosomal targeting of ATP7B, we employed immuno-electron microscopy (EM) analysis of ATP7B-GFP that exhibited trafficking and localization similar to the endogenous ATP7B (Figure S1D). In response to Cu, ATP7B-GFP moved from the tubular-vesicular TGN membranes (Figure 1F, arrows) to large multivesicular body (MVB)-like structures (Figure 1G, arrows; see also morphometry in Figure 1H), which contained numerous intraluminal vesicles (ILVs) and/or heterogeneous electron dense material (Figures 2A and 2B). These ultrastructural features allowed us to assign ATP7B-containing organelles to the LE/lysosome compartment (Saftig and Klumperman, 2009). Indeed, a double immunogold labeling revealed ATP7B-positive MVBs to contain LAMP1 (Figure 2B). Finally, we verified whether ATP7B is also transported to LE/lysosomal structures in vivo. Thin sections of mice liver revealed ATP7B-GFP (expressed via adenoviral vector) in MVB-like structures decorated by LAMP1 (Figure 2C) and similar to those observed in HepG2 cells. Therefore, ATP7B “vesicles” in the HepG2 line and in mouse hepatocytes can be defined as LE/lysosomes from both molecular and ultrastructural standpoints (for convenience, we will call them “lysosomes” through the rest of the manuscript).

Lysosomal localization of ATP7B prompted us to investigate whether the protein is directed to lysosomes for degradation that requires sorting into ILVs located in the lumen of lysosomes (Saftig and Klumperman, 2009). We found that only a small fraction of ATP7B was associated with ILVs and lysosome lumen (Figures 2A–2E), even when compared to LAMP1 (Figures 2A and 2E). Correspondingly, ATP7B levels remained unaffected when lysosome degradation was inhibited with bafilomycin A (Figure S2), indicating that ATP7B is targeted to lysosomes to perform a specific function at their limiting membranes, but not to be degraded.

### ATP7B Is Transported to the Lysosomal Compartment through a Direct Route that Emerges from the TGN

In response to Cu, ATP7B may travel via two possible routes: (1) it may first be delivered from the TGN to the cell surface and then be endocytosed to the lysosomes (indirect pathway) or (2) ATP7B might be conveyed from the TGN directly to the lysosomal compartments (direct pathway). To distinguish between these two possibilities, we treated HepG2 cells with tannic acid (TA), which blocks both the exo- and endocytic events at the level of the plasma membrane (Polishchuk et al., 2004). This treatment would prevent ATP7B trafficking to the lysosomes through the indirect pathway but would not impact the direct route. As a control, HepG2 cells were infected with the vesicular stomatitis virus (VSV) to express a thermosensitive t-45Os version of VSV glycoprotein (VSVG), a bona fide exocytic marker (Polishchuk et al., 2003). The cells were incubated at 20°C with BCS to accumulate both VSVG and ATP7B within the Golgi (Figure 2F). Cells were then shifted to 32°C in the presence of CuSO_4_ to activate both VSVG and ATP7B export from the TGN. In the absence of TA, VSVG was delivered from the TGN to the cell surface, whereas TA treatment caused VSVG arrest within TGN-derived transport carriers, which were docked at the plasma membrane (PM) but unable to fuse with acceptor membrane (Figure 2F). In contrast, accumulation of ATP7B within such post-Golgi VSVG-positive carries did not occur. Instead, most of ATP7B appeared within larger lysosome-like structures both in control and TA-treated cells (Figure 2F), indicating that ATP7B traffics directly from the TGN to lysosomes in response to an increase in Cu concentration.

To further verify this conclusion, we performed a time course analysis of ATP7B release from the TGN. As soon as 15 min after Cu addition, the ATP7B signal was detected in LAMP1-positive structures (Figure 2G, arrows). Such a fast rate of ATP7B trafficking argues against the indirect pathway because the uptake from the PM to lysosomes alone usually takes at least 30 min (Saftig and Klumperman, 2009). In addition, no ATP7B was observed at the surface of hepatocytes at that time point. Later (30 and 60 min after CuSO_4_ addition), the number of ATP7B-containing lysosomes progressively increased, whereas the Golgi area gradually lost the ATP7B signal (Figure 2G), supporting the direct transfer of ATP7B from the TGN to the lysosomal compartments.

We next investigated which Golgi-to-lysosome pathway is utilized by ATP7B. A large cohort of lysosomal proteins is carried from the TGN to endolysosomal compartments through transport events driven by clathrin and its adaptors, AP-1 and GGA (Saftig and Klumperman, 2009), whereas other lysosome residents (such as LAMP1 and MHC-II) take a clathrin-independent TGN-to-lysosome route (Pols et al., 2013; Saftig and Klumperman, 2009). We found that ATP7B did not associate with clathrin-coated profiles (Figure 1F, arrowheads) in the TGN area under neither low nor high Cu conditions. This is consistent with recent observations that neither AP-1 nor GGA suppression affects ATP7B export from the Golgi (Hirst et al., 2012). Further examination revealed ATP7B enrichment over the smooth TGN membrane domains (arrows in Figures 1F and 2H), which often contained LAMP1 (Figure 2H, arrows). Shortly after Cu stimulation, ATP7B was detected within 70–200 nm round or elongated membrane carriers, which occasionally exhibited internal membranes (Figure 2I, arrow) and therefore were similar to structures operating in direct Golgi-to-lysosome transport of LAMP1 (Pols et al., 2013). Indeed, these ATP7B carriers also frequently contained LAMP1 and were docked to the MVB-like structures (Figure 2I), indicating that ATP7B and LAMP1 may use the same pathway to travel from the TGN to lysosomal compartments.

### ATP7B Is Delivered from Lysosomes to Canalicular PM in Polarized Hepatocytes

ATP7B trafficking to lysosomes in response to Cu was unexpected and raised a question about the mechanism through which the lysosomes mediate Cu excretion from hepatocytes. One possibility would be that Cu efflux occurs through lysosomal exocytosis, a mechanism by which lysosomes fuse with the PM and secrete their content to the outside the cell (Andrews, 2000).

**Figure 3:**
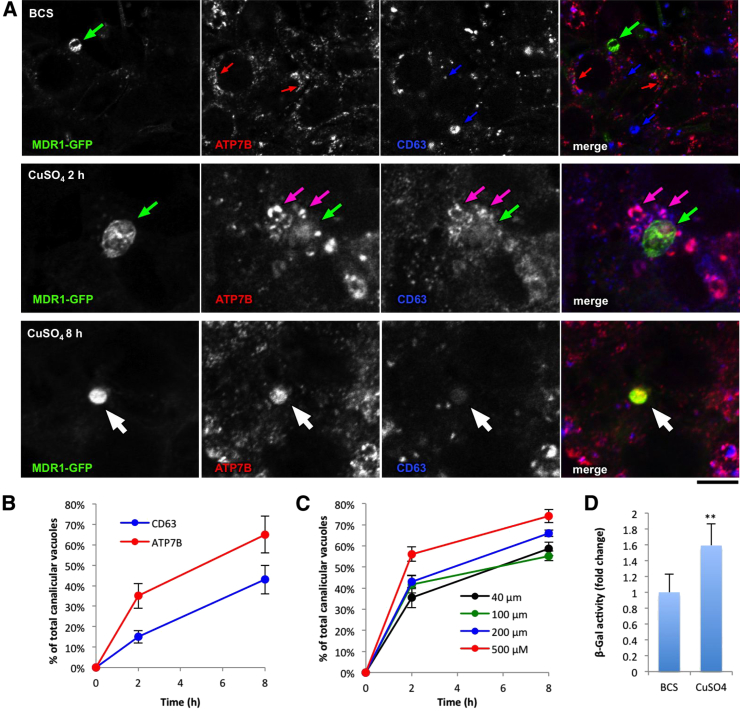
ATP7B Delivery to the Canalicular Domain of Polarized HepG2 Cells Requires a Lysosome Intermediate (A) Polarized HepG2-MDR1 cells were fixed directly after incubation with BCS or after additional treatment with CuSO_4_ for either 2 hr or 8 hr. After incubation with BCS, ATP7B was detected mainly within Golgi membranes (red arrows) but neither in CD63-positive lysosomes (blue arrows) nor in canalicular vacuoles (green arrows). Exposure to CuSO_4_ (2 hr) triggered ATP7B relocation to CD63-positive structures (pink arrows), which were frequently clustered around apical cysts (green arrows). White arrows in the lower row show canalicular vacuole, which received both ATP7B and CD63 after 8 hr incubation with CuSO_4_. (B) The percentage (mean ± SD; n = 20 fields) of ATP7B-positive or CD63-positive canalicular vacuoles increased in HepG2 cells over the time of incubation with CuSO_4_. (C) The cells were treated like in (A) with the exception that different CuSO_4_ concentrations were utilized. The percentage (mean ± SD; n = 20 fields) of ATP7B-positive canalicular vacuoles was calculated and plotted as a function of time. (D) Polarized HepG2-MDR1 cells were with BCS overnight or with CuSO_4_ for only 8 hr. The activity of β-Gal (mean ± SD; n = 3 experiments) in the canalicular cysts exhibits increase upon Cu stimulation. The scale bar represents 6.5 μm (A).

To examine whether ATP7B-containing lysosomes undergo apical exocytosis, we grew HepG2 cells under conditions that allowed for their polarization (Slimane et al., 2003). Upon polarization, neighboring hepatocytes form an apical (or biliary) cyst (vacuole) enriched in specific apical markers such as biliary salt transporters MDR1, MRP2, etc. (Slimane et al., 2003). Polarized HepG2 cells stably expressing canalicular marker MDR1-GFP (Slimane et al., 2003) were incubated with BCS to trap ATP7B within the Golgi and then exposed to 200 μM CuSO_4_ to follow the fate of ATP7B. In low Cu, ATP7B was mostly detected in the Golgi area (Figure 3A). Two hours after Cu stimulation, ATP7B exhibited a significant overlap with CD63 in lysosomes, which were frequently clustered around the biliary surface of the cells (Figure 3A). Notably, over 40% of apical cysts already exhibited ATP7B signal at this time point (Figure 3B). When incubation with CuSO_4_ was extended to 8 hr, the ATP7B labeling became more evident in canalicular cysts with 60% of them being ATP7B positive (Figures 3A and 3B). We also found that the redistribution of ATP7B to the canalicular domain of the cells occurred even upon moderate Cu increase (20–40 μM) and correlated with concentration of Cu and the duration of CuSO_4_ treatment (Figure 3C). A lower concentration of Cu (10 μM) was unable to induce ATP7B delivery to the canalicular membrane of HepG2 cells, although it still allowed for efficient ATP7B redistribution from the TGN to lysosomes (see Figure 1E).

Interestingly, we also detected CD63 together with ATP7B in the apical vacuoles upon Cu stimulation (Figures 3A and 3B). This suggests that ATP7B and CD63 were delivered together to the apical cysts of hepatocytes, likely through the induction of lysosomal exocytosis in response to Cu stimulation. Indeed, ATP7B delivery to the canalicular area of hepatocytes coincided with an increase in activity of lysosomal enzyme β-galactosidase (β-Gal) in biliary cysts upon Cu stimulation (Figure 3D).

Thus, apical lysosomal exocytosis may serve as a main route for Cu excretion in hepatocytes because it allows for (1) release of Cu from the ATP7B-positive lysosomal stores and (2) delivery of ATP7B to the canalicular domain.

### Modulation of Lysosomal Exocytosis Affects ATP7B Delivery to the PM

**Figure 4:**
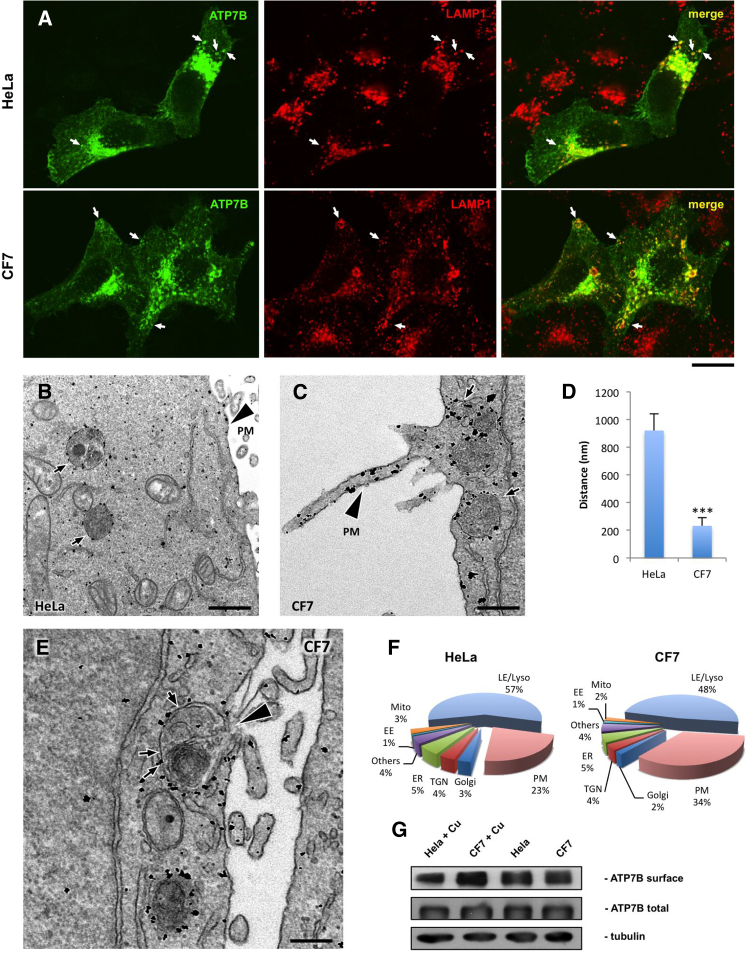
Activation of Lysosomal Exocytosis Stimulates Delivery of ATP7B to the Plasma Membrane (A) HeLa or CF7 cells were infected with adeno-ATP7B-GFP, incubated with CuSO_4_ for 2 hr, fixed, and stained for LAMP1. Arrows indicate ATP7B in lysosomes. (B and C) HeLa (B) or CF7 cells (C) were treated as in (A) and processed for immunogold EM to reveal ATP7B-GFP distribution. In both cell types, elevated Cu triggered ATP7B delivery to the lysosome-like structures (B and C, arrows) and plasma membrane (B and C, arrowhead). (D) Morphometry revealed reduction of the distance (mean ± SD; n = 100 lysosomes) between lysosomes and PM in CF7 cells. (E) Arrowhead indicates the site of fusion between ATP7B-positive lysosome (arrows) and PM in CF7 cell. (F) Pie plots exhibit percentage of ATP7B-associated gold particles in different compartments of HeLa and CF7 cells. (G) HeLa and CF7 cells were infected with adeno-ATP7B-GFP and then prepared for surface biotinylation directly or 2 hr after stimulation with 200 μM CuSO_4_. Western blot revealed higher amount of ATP7B at the surface of CF7 cells upon Cu increase. The scale bars represent 3.8 μm (A), 280 nm (B), 240 nm (C), and 220 nm (E).

Given that lysosomal exocytosis seems to be involved in the delivery of ATP7B to the cell surface, we decided to verify whether modulation of this process impacts ATP7B trafficking to the PM. To this end, we first used the CF7 HeLa cells that stably overexpress transcription factor EB (TFEB), a potent activator of lysosomal exocytosis (Medina et al., 2011). Stimulation with CuSO_4_ induced ATP7B redistribution from the Golgi to lysosomes and PM in both CF7 and parental HeLa cells (Figure 4A). CF7 cells exhibited ATP7B in numerous LAMP1-positive lysosomes positioned close to the peripheral regions of the cell membrane (Figure 4A). Such LAMP1-positive structures constitute a pool of peripheral lysosomes that actively undergo exocytosis (Medina et al., 2011). Indeed, EM revealed ATP7B in lysosome-like structures that were located significantly closer to the PM in CF7 cells than in control HeLa cells (Figures 4B and 4C, arrows, and 4D). Importantly, ATP7B-positive lysosomes were frequently seen to fuse directly with the PM in CF7 cells (Figure 4E), resulting in increase in the amount of ATP7B compared to the parental HeLa line (Figure 4F), as also confirmed by surface biotinylation (Figure 4G). Taken together, these observations suggest that TFEB-mediated activation of lysosomal exocytosis stimulates ATP7B delivery to the cell surface under high Cu conditions.

**Figure 5:**
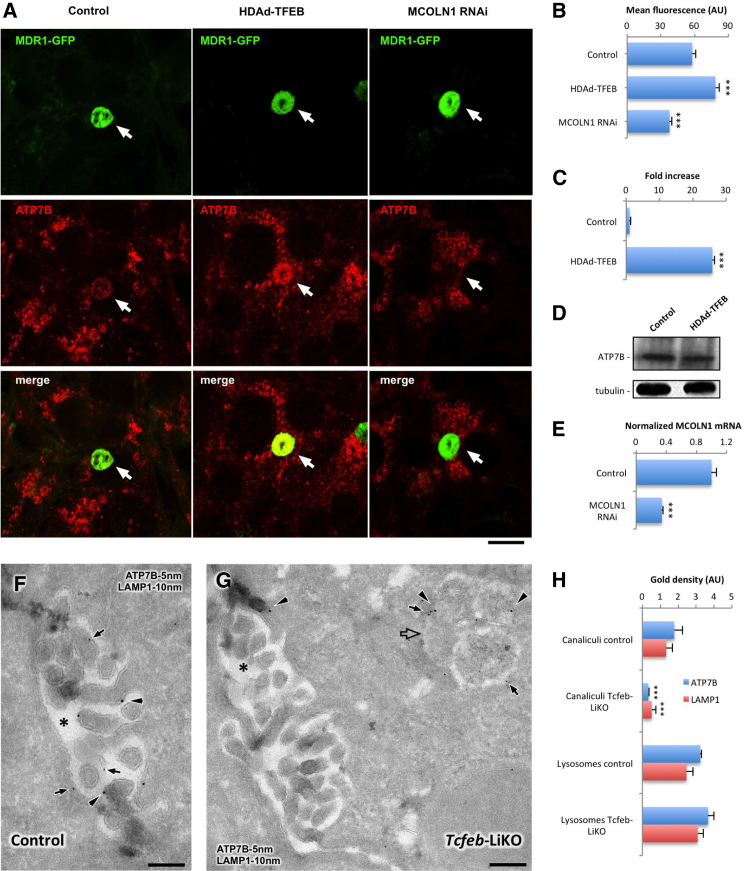
Modulation of Lysosomal Exocytosis Impacts ATP7B Delivery to the Canalicular Domains of Hepatic Cells In Vitro and In Vivo (A) Polarized HepG2-MDR1 cells were infected with HDAd-TFEB (middle column) or incubated with MCOLN1-specific small interfering RNAs (siRNAs) (right column) and exposed to 200 μM CuSO_4_ for 8 hr. Immunofluorescent labeling of endogenous ATP7B revealed increase in its amount in the area of canalicular vacuoles (arrows in the midcolumn) in the cells infected with HDAd-TFEB and decrease in the canalicular area (arrows in the right column) of MCOLN1-silenced cells. (B) Quantification shows ATP7B-associated fluorescence in the MDR1-positive canalicular vacuoles (mean ± SD; n = 50 canalicular vacuoles). (C) qRT-PCR indicated increase in TFEB mRNA levels in the cells infected with virus containing TFEB DNA. (D) Western blot revealed that total amounts of endogenous ATP7B remained similar in control and TFEB-overexpressing cells. (E) qRT-PCR shows decrease in MCOLN1 mRNA levels in MCOLN1-silenced cells. (F and G) Liver tissue from the control and *Tcfeb*-LiKO mice injected with adeno-ATP7B-GFP were prepared for cryo-immuno-EM, which revealed ATP7B (F, arrows) and LAMP1 (F, arrowheads) in the canalicular region (F, asterisk) in control mice. LAMP1 (G, arrowheads) and ATP7B (G, arrows) exhibited poor signal at the canalicular membrane (G, asterisk) in *Tcfeb*-LiKO mice but were detected in neighbor lysosome (G, open arrow). (H) ATP7B and LAMP1 labeling densities were calculated in canalicular domains (mean ± SD; n = 50 canalicular areas) and in lysosomes (mean ± SD; n = 50 lysosomes). The scale bars represent 3.5 μm (A), 240 nm (F), and 270 nm (G).

To test whether this is also the case in a liver-relevant cell system, polarized MDR1-GFP HepG2 cells were infected with a helper-dependent adenovirus carrying TFEB DNA (HDAd-TFEB) (Figures 5A–5D), which resulted in an increase in TFEB expression (Figure 5C). TFEB- and mock-infected cells were then exposed to CuSO_4_, and the ATP7B signal in MDR1-GFP-positive biliary cysts was analyzed. We found that overexpression of TFEB resulted in a higher amount of ATP7B delivered to the apical domain of HepG2 cells (Figures 5A and 5B), whereas the total quantity of ATP7B remained the same (Figure 5D).

In a parallel series of experiments, we inhibited lysosomal exocytosis by suppressing mucolipin-1 (MCOLN1) using RNAi. MCOLN1 is a Ca^2+^ channel, which promotes lysosome fusion with PM (Medina et al., 2011). Reduction of MCOLN1 expression in HepG2 cells (Figure 5E) resulted in a significant decrease in ATP7B delivery to biliary surface (Figures 5A and B). Given that Ca^2+^ is required for lysosomal exocytosis (Andrews, 2000), we used the Ca^2+^ chelator BAPTA as another tool to inhibit lysosomal exocytosis and observed reduction in ATP7B trafficking to the canalicular domain in BAPTA-treated cells (not shown).

Finally, we employed a mice model with a liver-specific knockout of *TFEB* (*Tcfeb*-LiKO mice) (Settembre et al., 2013) to evaluate whether inhibition of TFEB-mediated lysosomal exocytosis affects delivery of ATP7B to the canalicular sites of hepatocytes in vivo. To this end, control and *Tcfeb*-LiKO mice were given Cu in their drinking water as described (Gross et al., 1989). The mice were sacrificed 4 hr after stimulation with Cu and their livers processed for analysis. EM revealed specific ATP7B and LAMP1 signals in canalicular area of hepatocytes in the liver of control mice (Figure 5F), indicating efficient delivery of both proteins from the lysosomes upon Cu overload. The presence of ATP7B and LAMP1 at the canalicular membrane of hepatocytes correlated with increased activity of β-Gal and β-hexosaminidase (β-Hex) in the bile (Figure S3A). This suggests that Cu stimulates lysosomal exocytosis at the biliary surface of hepatocytes and thus facilitates ATP7B delivery from lysosomal structures to the canalicular membrane.

In contrast, *TFEB* deletion in the liver of *Tcfeb*-LiKO mice resulted in significant reduction of both ATP7B and LAMP1 labeling at the biliary membrane of hepatocytes (Figures 5G and 5H). We reasoned that the decrease of ATP7B at the canalicular domains in *Tcfeb*-LiKO mice liver was due to the suppression of lysosomal exocytosis. We found that ATP7B/LAMP1-positive lysosomes can be detected near canaliculi (Figure 5G) in *Tcfeb*-LiKO mice and that ATP7B/LAMP1 labeling densities in such lysosomes were similar to those in control animals (Figure 5H). However, deletion of *Tcfeb* did not allow ATP7B lysosomes to fuse with apical membrane of hepatocytes and therefore to convey ATP7B to the canalicular surface. This correlated with a significant reduction of β-Hex and β-Gal activities in the bile of *Tcfeb*-LiKO mice despite Cu stimulation (Figure S3B).

Thus, taken together, both in vitro and in vivo observations support the involvement of lysosomal exocytosis in targeting ATP7B to the biliary surface domain in hepatic cells.

### Activation of Lysosomal Exocytosis Increases Copper Excretion from the Cells

**Figure 6:**
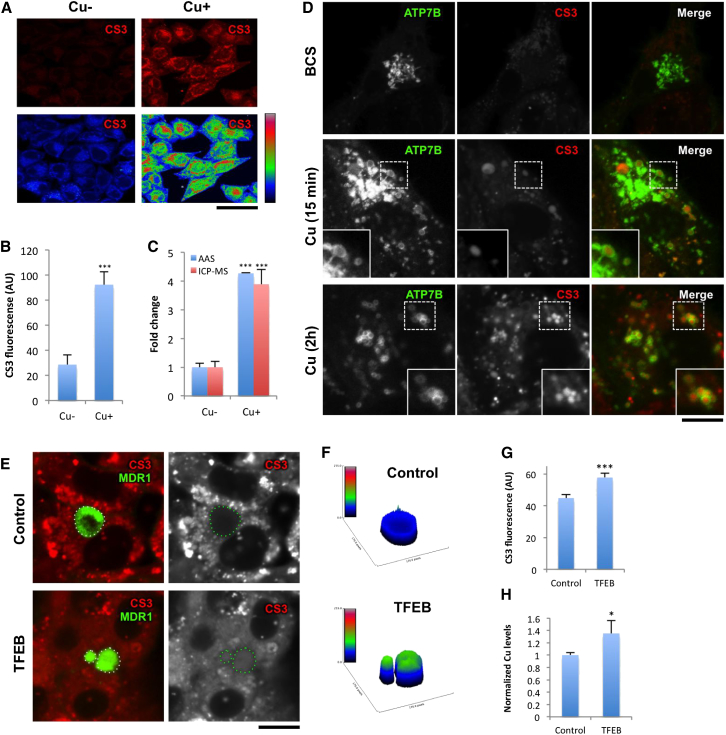
Activation of Lysosomal Exocytosis Stimulates Cu Excretion from HepG2 Cells (A) HepG2 cells were incubated with either 200 μM BCS or 200 μM CuSO_4_ for 2 hr loaded with CS3 for 15 min before visualization at the confocal microscope. Cu-associated CS3 signal was low in BCS-treated cells but significantly increased in Cu-loaded cells (see false color images of CS3 intensity in lower row). (B) Quantification of the CS3 fluorescent intensity (mean ± SD; n = 15 fields) indicated its increase in CuSO_4_-treated cells. (C) Cells were treated like in (A) and prepared for atomic adsorption spectroscopy (AAS) or inductively coupled plasma mass spectrometry (ICP-MS), which revealed an increase in intracellular Cu concentration (mean ± SD; n = 4 experiments) in CuSO_4_-treated cells. (D) HepG2 cells were infected with adeno-ATP7B-GFP, exposed to BCS, and observed in confocal microscope either directly or after 15 min or 2 hr incubation with CuSO_4_. Insets show CS3 fluorescence within circular ATP7B lysosomes. (E) Polarized HepG2-MDR1 cells were infected with HDAd-TFEB and then exposed to 200 μM CuSO_4_ for 8 hr and loaded with CS3. (F) 3D plots show the intensity of the CS3 signal in the corresponding canalicular areas (dash line in D). (G) Levels of CS3 fluorescence within canalicular cysts of HepG2 cells (mean ± SD; n = 100 cysts) increased in cells overexpressing TFEB. (H) Polarized HepG2-MDR1 cells were infected with HDAs-TFEB and treated with CuSO_4_ as in (E). Afterward, canalicular cysts were opened with EDTA and their content was analyzed for Cu using ICP-MS. Normalized Cu concentration (mean ± SD; n = 3 experiments) increased in the biliary cysts of cells overexpressing TFEB. The scale bars represent 7.5 μm (A) and 4 μm (D and E).

To further test the impact of lysosomal exocytosis on Cu homeostasis, we investigated whether this process is involved in the regulation of Cu efflux from liver cells. Coppersensor 3 (CS3) was employed to analyze intracellular levels of exchangeable Cu (Dodani et al., 2011). Variations of intracellular Cu levels observed in the control experiments with CS3 (Figures 6A and 6B) were confirmed by spectroscopy (Figure 6C).

Then, we investigated subcellular distribution of CS3 in cells expressing ATP7B-GFP. HepG2 cells treated with BCS exhibited low CS3 signal in the cytoplasm, whereas ATP7B-GFP was mainly detected in the Golgi area (Figure 6D). Shortly (15 min) after exposing cells to Cu, ATP7B appeared in the lysosomes where increased CS3 signal was detected (Figure 6D). Longer incubation with CuSO_4_ (up to 2 hr) induced a complete redistribution of ATP7B to the lysosomes (Figure 6D), where CS3 fluorescence further concentrated, indicating that ATP7B lysosomes could be used for temporary Cu storage/sequestration.

Next, we reasoned that activation of lysosomal exocytosis should allow reduction of intracellular Cu due to release of the metal from the lysosome into canalicular vacuoles. To test this, we used polarized HepG2 cells, which were infected with HDAd-TFEB to activate lysosomal exocytosis, exposed to CuSO_4_, and labeled with CS3. Confocal microscopy revealed the CS3 signal to be higher in biliary cysts of TFEB-overexpressing cells than in those of control cells (Figures 6E–6G). Meanwhile, CS3 fluorescence decreased in the cytoplasm of TFEB-infected cells (Figure 6E). Elevation of Cu levels in the apical vacuoles of TFEB-overexpressing HepG2 cells was confirmed by inductively coupled plasma mass spectrometry (ICP-MS) (Figure 6H), indicating that stimulation of lysosomal exocytosis helps to excrete Cu from hepatocytes into biliary areas.

### ATP7B Silencing Inhibits Apical Lysosomal Exocytosis, Cu Excretion, and Polarization of Hepatic Cells

**Figure 7:**
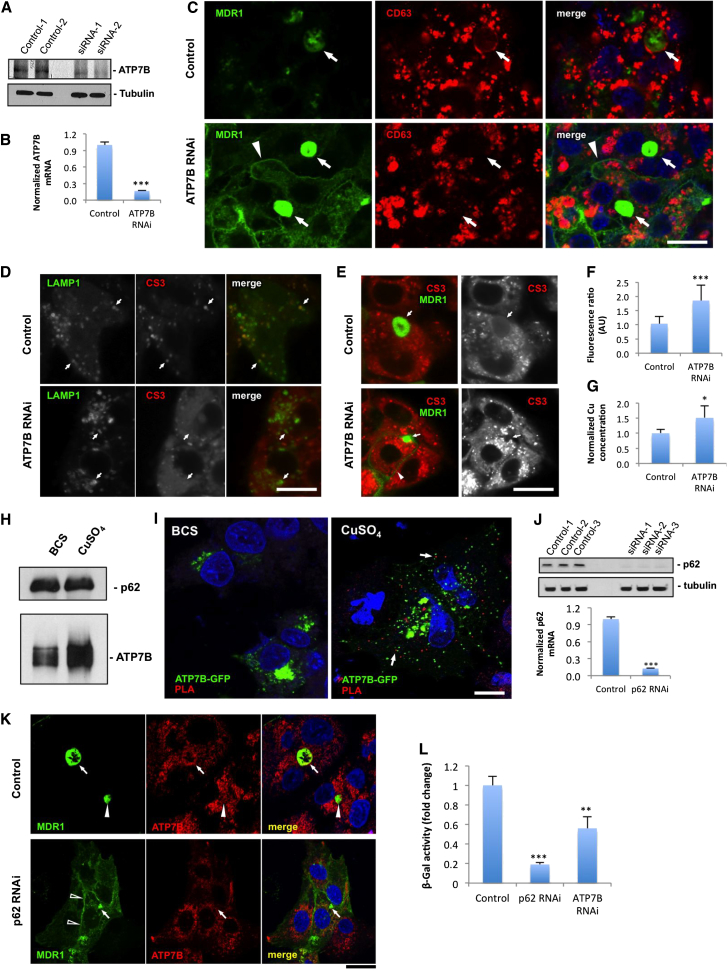
ATP7B Regulates Lysosomal Exocytosis and Cu Excretion through Interaction with p62 (A) Western blot reveals reduction in ATP7B expression in HepG2-MDR1 incubated with ATP7B-specific siRNAs. (B) qRT-PCR indicates reduction of ATP7B mRNA levels (mean ± SD; n = 3 experiments) in ATP7B-silenced cells. (C) Polarized control and ATP7B-silenced HepG2-MDR1 cells were exposed to 200 μM CuSO_4_ for 8 hr, fixed, and stained for CD63. Arrows indicate apical cysts whereas arrowheads show MDR1 mistargeting from the canalicular area. (D) Control and ATP7B-silenced HepG2 cells were transfected with LAMP1-GFP, exposed to CuSO_4_, and loaded with CS3. Arrows indicate LAMP1-positive structures. (E) Control and ATP7B-silenced HepG2-MDR1 cells were treated with 200 μM CuSO_4_ for 8 hr and loaded with CS3. Arrows show MDR1-positive canalicular vacuoles. (F) Ratio between intracellular and canalicular CS3 fluorescence (mean ± SD; n = 100 cells) increased in ATP7B-deficient cells. (G) Cells were treated like in (E) and prepared for ICP-MS, which revealed an increase in normalized Cu concentration (average ± SD; n = 4 experiments) upon ATP7B depletion. (H) HepG2 cells were infected with adeno-ATP7B-GFP, exposed to BCS and or CuSO_4_, lysed, and subjected to immunoprecipitation with anti-p62 antibody. Western blot reveals that similar amount of p62 pulls down higher amount of ATP7B in CuSO_4_-treated cells. (I) The cells were infected and treated like in (A) and processed for PLA analysis (see Experimental Procedures). PLA signal indicating close association of ATP7B and p62 was detectable as red spots (arrows) only in CuSO_4_-treated cells. (J) Western blot and qRT-PCR indicate reduction of p62 at both protein and mRNA levels in HepG2-MDR1 cells incubated with p62-specific siRNAs. (K) Control and p62-silenced polarized HepG2-MDR1 cells were exposed to CuSO_4_, fixed, and stained for endogenous ATP7B. Arrows indicate canalicular cysts. Empty arrowheads indicate MDR1 mistargeting from the canalicular area. (L) Control, p62-silenced, or ATP7B-silenced HepG2 cells were exposed to CuSO4 for 8 hr. The chart shows decrease of normalized activity of β-Gal (mean ± SD; n = 3 experiments) in the canalicular cysts upon depletion of either p62 or ATP7B. The scale bars represent 4 μm (C–E), 3 μm (I), and 5.2 μm (K).

We then determined whether ATP7B is required for lysosome exocytosis at the apical surface of hepatic cells. To this end, we silenced ATP7B expression in polarized HepG2 cells (Figures 7A and 7B) and found that, in contrast to control cells, ATP7B-deficient cells exhibited no CD63 within canalicular domains (Figure 7C, arrows). This finding suggests that ATP7B presence at the lysosomes might define their ability to undergo apical exocytosis. Interestingly, ATP7B ablation also affected polarization of HepG2 cells, as we detected a reduction in the number of hepatocytes making MDR1-positive canalicular cysts (Figures S4A, arrows, and S4B) and a mistargeting of MDR1 to the basolateral surface in silenced cells (Figures 7C, arrowheads, and S4A).

We also investigated the intracellular distribution of Cu in ATP7B-deficient cells. We detected CS3 fluorescence within the LAMP1-GFP-positive structures of control cells, whereas in ATP7B-silenced cells, CS3 was hardly visible in LAMP1-GFP spots (Figure 7D, arrows), indicating that lysosomes did not receive Cu in the absence of ATP7B. Finally, we also found that CS3 fluorescence in the canalicular area of ATP7B-deficient HepG2 cells was lower than in control hepatocytes (Figure 7E, arrows), whereas intracellular CS3 signal increased (Figures 7E and 7F). Elevated intracellular Cu levels in ATP7B-silenced HepG2 cells were also confirmed by ICP-MS (Figure 7G).

Taken together, these results indicate that ATP7B is involved (1) in Cu import into lysosomes and (2) in apical exocytosis of such lysosomes that allows elimination of excess Cu from the cells and supports hepatocyte polarity.

### Cu-Dependent Interaction with p62 Dynactin Subunit Defines ATP7B Targeting to the Canalicular Surface of Hepatic Cells

To understand the molecular mechanism through which ATP7B targets lysosomes to the apical surface of hepatocytes, we analyzed the publications on Cu-dependent protein interactions of ATP7B. From this information, we found the interaction between ATP7B and the p62 subunit of dynactin (DNCT4) to be particularly attractive from the trafficking standpoint (Lim et al., 2006). Given that the minus ends of the microtubules are oriented toward the canalicular domain of hepatocytes (Cohen et al., 2004), the binding to p62 may allow ATP7B-containing membranes to anchor the dynein motor and, therefore, be translocated to the biliary surface.

To test this hypothesis, we first immunoprecipitated endogenous p62 from either BCS- or CuSO_4_-treated HepG2 cells and found significantly higher amounts of ATP7B in pull-downs from the cells kept in high Cu (Figure 7H). Cu-dependent association of ATP7B with p62 was confirmed further using proximity ligation assay (PLA) (D’Agostino et al., 2013). Figure 7I shows a clear PLA signal, indicating close association between ATP7B and p62 in cells that were exposed to CuSO_4_. Such PLA signal was lacking in BCS-treated cells (Figure 7I), suggesting that the effective interaction of ATP7B with p62 occurs only when the intracellular Cu increases. We then analyzed the impact of p62 silencing (Figure 7J) on Cu-dependent trafficking of ATP7B. We found that p62 depletion did not affect ATP7B transport from the Golgi to lysosomes upon increase in Cu concentration (Figure S4C). However, further clustering of ATP7B-contaning lysosomes around the apical cyst and delivery of ATP7B to the canalicular surface were seriously compromised in p62-deficient HepG2 cells (Figure 7K, arrows). To further evaluate the impact of p62-ATP7B interaction on the lysosomal exocytosis, we depleted p62 or ATP7B and measured the activity of lysosomal enzyme β-Gal within canalicular cysts of polarized HepG2 cells stimulated with CuSO_4_. Both ATP7B-silenced and p62-silenced cells exhibited significant reduction of enzyme activities within the canalicular vacuoles (Figure 7L).

Notably, as it occurred in ATP7B-depleted cells, ablation of p62 resulted in partial loss of polarity of HepG2 cells and partial missorting of MDR1 from canalicular cysts (Figures 7K, arrowheads, S4D, and S4E). In contrast, the distribution of basolateral markers such as E-cadherin and Na/K-ATPase remained intact (Figure S4B) in silenced cells.

Taken together, these findings suggest (1) that the Cu-dependent interaction between ATP7B and p62 is required for apical exocytosis of ATP7B-positive lysosomes at the canalicular surface of hepatocytes and (2) that this process contributes to the polarization of HepG2 cells.

### Activation of Lysosomal Exocytosis Accelerates Cell Surface Delivery of the Most Frequent Wilson-Disease-Causing ATP7B Mutant

**Figure 8:**
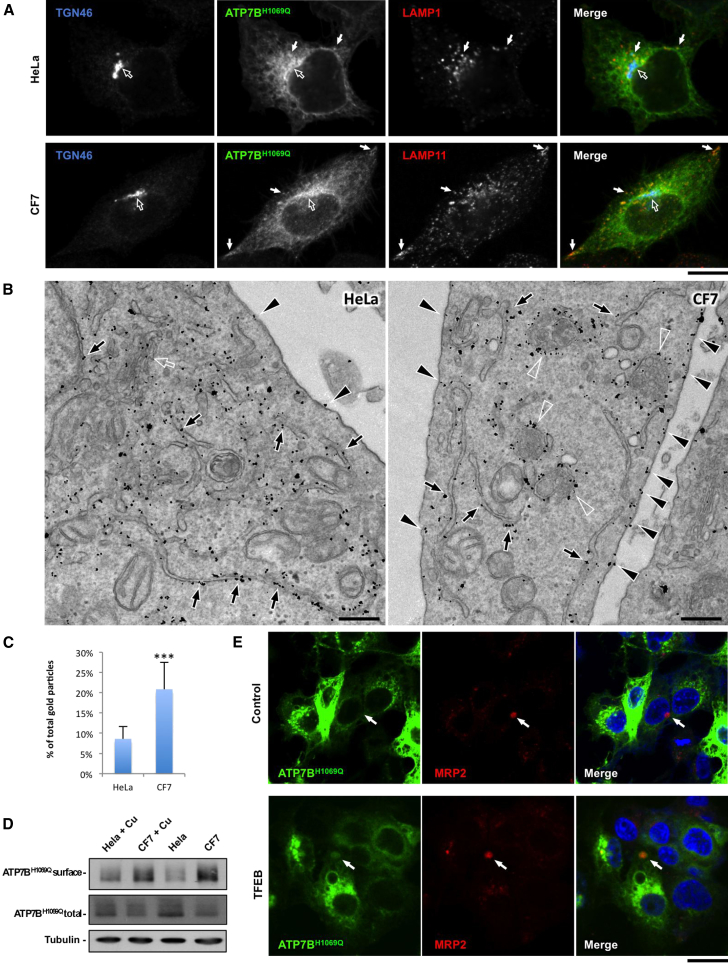
Activation of Lysosomal Exocytosis Improves Delivery of Most-Frequent WD-Causing ATP7B^H1069Q^ Mutant to the Cell Surface (A) Control HeLa cells and CF7 cells were infected with adeno-ATP7B^H1069Q^-GFP, incubated with 200 μM CuSO_4_ for 2 hr, and stained for LAMP1 and TGN 46. Open and solid arrows show Golgi and lysosomes, respectively. (B) Control HeLa and CF7 cells were treated as in (A), fixed, and processed for immunogold EM to reveal ATP7B^H1069Q^ distribution. Although ATP7B^H1069Q^ was mistargeted to the ER (arrows), it can be detected also in the Golgi (empty arrow) and lysosomes (empty arrowheads). Filled arrowheads indicate higher amount of ATP7B^H1069Q^ at the surface of CF7 cells. (C) Quantification revealed increase in the percentage of ATP7B-associated gold particles (average ± SD; n = 30 cells) at the plasma membrane in CF7 cells. (D) HeLa and CF7 cells were infected with adeno-ATP7B^H1069Q^-GFP and then prepared for surface biotinylation directly or 2 hr after stimulation with 200 μM CuSO_4_. Western blot revealed higher amount of ATP7B at the surface of CF7 cells. (E) Polarized HepG2 cells expressing ATP7B^H1069Q^-GFP were infected with HDAd-TFEB, incubated with 200 μM CuSO_4_ for 8 hr, and stained with canalicular marker MRP2. Arrows indicate canalicular cyst. The scale bars represent 3.8 μm (A), 240 nm (B), and 6.5 μm (E).

Finally, we determined whether activation of lysosomal exocytosis could be utilized as a therapeutic strategy to contrast Wilson disease (WD) pathogenesis. The most frequent ATP7B mutant H1069Q (up to 50% in Caucasian population; Payne et al., 1998), exhibits residual catalytic activity (van den Berghe et al., 2009) but is retained within the endoplasmic reticulum (ER), where it undergoes degradation (Payne et al., 1998; van den Berghe et al., 2009). Thus, correction of this mutant to the regular functional compartment might be beneficial for the large cohort of the WD patients. Interestingly, despite extensive retention within the ER (Figures 8A and 8B, arrows), some ATP7B^H1069Q^ gets transported to the Golgi (empty arrows in Figures 8A and 8B) and further to LAMP1-positive structures (Figure 8A, solid arrows). Thus, we reasoned that acceleration of lysosomal exocytosis might allow a more-efficient supply of residual ATP7B^H1069Q^ to the cell surface, where it can transport Cu out of the cell. To test this, TFEB-overexpressing CF7 cells were infected with an adenovirus carrying ATP7B^H1069Q^ and exposed to CuSO_4_. Arrows in Figure 8A show that exocytosis-prone lysosomes, which reside near the surface of CF7 cells (Medina et al., 2011), received ATP7B^H1069Q^. This coincided with a stronger immunogold labeling of the mutant protein at the surface of CF7 cells compared to the parental HeLa line (Figures 8B, arrowheads, and 8C). Correspondingly, a biotinylation assay revealed a significant increase in the amount of ATP7B^H1069Q^ at the surface of CF7 cells (Figure 8D). Therefore, activation of lysosomal exocytosis allowed recovery of additional quantities of ATP7B^H1069Q^ at the cell surface.

To verify this conclusion in a liver-relevant system, polarized HepG2 cells expressing ATP7B^H1069Q^ were infected with HDAd-TFEB and exposed to CuSO_4_ for 8 hr. Confocal microscopy revealed that, in control cells, ATP7B^H1069Q^ was hardly detectable within the MRP2-positive canalicular cysts, whereas overexpression of TFEB stimulated delivery of the mutant ATP7B toward the canalicular domain of hepatocytes (Figure 8E, arrows). Therefore, activation of lysosomal exocytosis allows recovery of additional amounts of ATP7B^H1069Q^ at the cell surface.

## Discussion

Our findings indicate that exposure of hepatocytes to increasing Cu concentrations induces ATP7B trafficking from the TGN to subset of lysosomes, where ATP7B imports Cu into the lysosomal lumen and where the metal can be transiently stored. Further Cu increase over a threshold value (approximately 20 μM) induces the exocytosis of lysosomes containing ATP7B with subsequent delivery of the Cu transporter to the apical surface of hepatocytes and the release of Cu into the biliary space. Importantly, ATP7B determines both the ability of the lysosome to undergo exocytosis and also the apical/canalicular direction of the exocytic process. Apparently, exocytosis is triggered by Cu-dependent interaction of ATP7B with p62 (DNCT4), which allows ATP7B to anchor lysosomes on microtubule highways directed toward the apical pole of hepatocytes. In our view, this sequence of events outlines the main mechanism, which is utilized by hepatocytes to remove excess Cu from liver and which is affected by ATP7B mutations in Wilson disease.

Some signs of this mechanism were uncovered more than 20 years ago when lysosomes were suggested to operate in Cu homeostasis (Gross et al., 1989). Later, ATP7B was even detected in late endosomes (Harada et al., 2000, 2005), and its involvement in the secretion of some lysosomal enzymes into bile was reported (Sugawara et al., 1995). Unfortunately, the conclusive experiments that would directly demonstrate a role of lysosomal exocytosis in ATP7B trafficking, molecular mechanisms behind this process, and its coordination with Cu excretion were not performed. Therefore, above findings remained mostly neglected over the last decade. This happened mainly because ATP7B trafficking and compartmentalization, which constitutes the centerpiece of Cu homeostasis in liver, remained poorly understood and highly controversial (La Fontaine and Mercer, 2007; Polishchuk and Lutsenko, 2013). The major mystery in the field was the nature of so-called “vesicles,” where ATP7B resides in high-Cu conditions and how such vesicles operate in Cu excretion.

In this study, identification of the ATP7B transport itinerary allowed us to close these gaps and to complete the puzzle of the mechanism at the basis of Cu excretion in hepatocytes. Our initial finding revealed a subpopulation of lysosomes as the main intermediate in ATP7B trafficking. We demonstrated lysosomes to receive ATP7B directly from the TGN in response to increasing Cu and to actively use this pump for Cu import. This allowed us to assign the elusive “vesicular” ATP7B compartment with identity of the lysosomes.

The lysosomal localization of ATP7B in high Cu conditions raises several issues regarding the ATP7B-dependent mechanisms of Cu homeostasis. The first is whether Cu is required in the lysosome or it is merely sequestered there. Cu in lysosomes can be utilized as a cofactor by housekeeping enzymes (acid sphingomyelinase) (Qiu et al., 2003). Interestingly, another Cu pump, ATP7A, supplies the metal in a similar way to tyrosinase across the membrane of lysosome-related organelles melanosomes (Setty et al., 2008). ATP7A was also found to transport Cu into LAMP1/Rab7-positive phagosomes of macrophages, where the metal has been hypothesized to kill engulfed bacteria (White et al., 2009). Thus, the ability to reach the lysosomal compartment and to function there could be a common feature of both Cu ATPases. Importantly, low pH in the lysosomes does not inhibit metal-transporting activity of ATP7B but favors it (Safaei et al., 2008).

The second issue is whether liver lysosomes may operate as Cu storage compartments. Our data suggest that lysosomes can uptake excess Cu from the cytosol through ATP7B (Figure 6), whereas the release of Cu in the opposite direction could occur through the lysosome-targeted Cu channel CTR2 (van den Berghe et al., 2007) when this metal is needed in the cytosol. We found that, at the Cu concentrations below 20 μM, ATP7B reaches the lysosomes from the TGN, but these lysosomes do not undergo exocytosis unless Cu levels increase further. This probably allows the ATP7B lysosomes to transiently store Cu, when its concentration does not yet threat cell homeostasis. Therefore, Cu fluxes to and from the lysosomes and, hence, Cu storage in these organelles should be tightly regulated. Indeed, yeast cells utilize vacuole (lysosome analog) for Cu storage (Nevitt et al., 2012), indicating that this function of lysosomes is conserved in evolution.

The third issue is whether an ATP7B-positive subset of lysosomes resembles lysosome-related organelles (LROs), which discharge their content in response to specific stimuli (Raposo et al., 2007). This property may be required for rapid release of Cu from the lysosome lumen into the bile in response to Cu overload. However, we found that neither Rab27A nor VAMP7 nor synaptotagmin7 depletion affected exocytosis of ATP7B-positive structures (Figure S5). Thus, in terms of exocytosis, ATP7B-positive lysosomes do not resemble LROs, which require Rab27A (Raposo et al., 2007). On the other hand, ATP7B-positive lysosomes share only some common elements of the exocytic molecular machinery (Ca^2+^ and MCOLN1) with common lysosomes, which need VAMP7 and synaptotagmin7 for exocytosis. In our view, the lysosomes may require a specific and unique asset of molecules for apical exocytosis in hepatocytes. ATP7B itself may be a part of such specific machinery, as it is expressed almost exclusively in hepatic cells (Lutsenko et al., 2007).

In this context, another significant finding of our study indicates lysosomal exocytosis to operate for both ATP7B and Cu delivery to the biliary surface of hepatocytes. Lysosomal exocytosis plays a major role in several physiological processes such as cellular immune response, bone resorption, and PM repair (Andrews, 2000). Our in vitro and in vivo data suggest that stimulation of lysosomal exocytosis increases both ATP7B and Cu in biliary cysts. Thus, it turns out that exocytosis of the lysosomes allows hepatocytes to expel sequestered Cu and to deliver ATP7B to canalicular surface, where it might pump Cu directly from the cytosol into the bile (Hubbard and Braiterman, 2008).

Our discovery of the ATP7B trafficking mechanism poses new questions. The first question addresses the way in which Cu triggers exocytosis of the lysosomes that receive ATP7B. We found that ATP7B ablation inhibits lysosome clustering and the release of lysosomal content at the biliary surface (Figures 7C and 7L). In line with these observations, ATP7B-deficient rats exhibit significant decrease of lysosome enzyme activity in the bile (Sugawara et al., 1995), presumably due to suppression of lysosomal exocytosis in the absence of functional ATP7B. Thus, the presence of ATP7B probably defines whether a given lysosome has to undergo exocytosis in hepatocytes when Cu increases. Moreover, ATP7B also determines the apical/canalicular direction of such exocytosis. We found that the ability of ATP7B to drive exocytic processes in response to Cu is likely governed by ATP7B’s interaction with the p62. p62 interacts with ATP7B in the presence of high Cu (Lim et al., 2006; see also Figures 7H and 7I) and therefore, being in complex with dynactin and dynein motor, can probably pull ATP7B-enriched lysosomes to the microtubule minus ends, which are oriented toward the canalicular domain of hepatocytes (Cohen et al., 2004). Indeed, we observed that depletion of p62 does not allow ATP7B-containing lysosomes to move toward the apical pole of HepG2 cells and to deliver ATP7B to the canalicular surface, even in the presence of excess Cu (see Figure 7). These findings are in line with previous studies showing microtubule disruption to impair delivery of ATP7B to the canalicular surface of HepG2 cells (Roelofsen et al., 2000). Our observations also indicate that ATP7B-mediated lysosomal exocytosis may contribute to polarization of hepatic cells (Figure S4), likely facilitating the delivery of specific proteins and lipids to the apical membrane domain. Partial disorganization of liver architecture and hepatic tumor development in ATP7B-deficient mice (Huster et al., 2006) argues in favor of this hypothesis.

The second question is whether and how the lysosome exocytosis pathway may be utilized for therapeutic purposes. Stimulation of lysosomal exocytosis via TFEB overexpression has already been shown to promote cellular clearance in lysosomal storage diseases (Medina et al., 2011). Here, we found that transcriptional activation of lysosomal exocytosis allows partial recovery of the proper subcellular localization of the most frequent WD-causing mutant to the regular ATP7B functional site. Given that this mutant possesses significant residual Cu-transporting activity (van den Berghe et al., 2009), its rescue to the correct location on the biliary surface of hepatocytes could be beneficial for a large cohort of WD patients. On the other hand, toxic Cu accumulation in lysosomes has been reported during pathogenesis of choleostatic disorders (Gross et al., 1989) and could be probably circumvented (contrasted) through activation of the lysosome fusion with the cell membrane. In addition, the well-known role of ATP7B in Wilson disease has been recently expanded to its involvement in other pathologies such as modulation of the Alzheimer’s disease phenotype and anticancer drug resistance (Gupta and Lutsenko, 2009). Thus, the ATP7B-dependent lysosomal exocytosis emerges as a promising therapeutic target to combat WD and a number of other disorders.

## Experimental Procedures

### Cell Culture and Transfection and Construction of Recombinant Adenoviruses

HepG2, HepG2-MDR1, HeLa, and HeLa CF7 cells were grown in Dulbecco’s modified Eagle’s medium supplemented with 10% fetal calf serum (decomplemented for HepG2), 2 mM L-glutamine, penicillin, and streptomycin. For transfection of plasmids, jetPEI TM-Hepatocyte (Polyplus transfection) and Trans IT LT1 (Tema Ricerca SRL) transfection reagents were used for HepG2 and HeLa, respectively.

### Trafficking Assay and Cu Treatment

To investigate localization of ATP7B at the different Cu load, cells were treated with 200 μM Cu-chelating agent BCS and with different concentrations of CuSO_4_. To compare trafficking of ATP7B with VSVG, cells were incubated with BCS (overnight), then infected with VSV (Polishchuk et al., 2003), incubated at 20°C in the presence of BCS (to accumulate both proteins in the Golgi), and finally warmed to 32°C and treated with CuSO_4_ (to activate post-Golgi transport of both proteins). Tannic acid (0.5%) was added in some experiments during release of 20°C block to inhibit fusion of post-Golgi transport carriers with the PM (Polishchuk et al., 2004).

### Mice and Treatment

Two-month-old males of *Tcfeb*-flox mouse (Settembre et al., 2013) were used. TcFEB loxP/loxP mice that did not carry the albumin Cre were utilized as a control. To express ATP7B-GFP, mice were subjected to retro-orbital injection with adeno-ATP7B-GFP 3 days before the experiment. To stimulate Cu excretion from liver, both control and *Tcfeb*-flox mice received 0.125% CuSO_4_ in water 4 hr before the animals were sacrificed (Gross et al., 1989). Liver tissue was rapidly dissected from the mice, fixed, and processed for electron microscopy, whereas bile was collected from gall bladder for β-Gal and β-Hex assays. All experiments were approved by the Committee on Animal Care at Baylor College of Medicine and conform to the legal mandates and federal guidelines for the care and maintenance of laboratory animals.

### Immunofluorescence and CS3 Labeling

Cells were fixed for 10 min with 4% paraformaldehyde in 0.2 M HEPES, permeabilized, labeled with primary and secondary antibodies, and examined with a ZEISS LSM 700 or LSM 710 confocal microscope equipped with a 63× 1.4 numerical aperture oil objective. For fluorescent Cu detection, cells were incubated with 5 μM CS3 solution for 15 min at 37°C. CS3 was excited with 561 nm laser of LSM710, and its emission was collected from 565 to 650 nm. Colocalization module of ZEISS ZEN 2008 software was used to measure colocalization of ATP7B with different intracellular markers. ATP7B fluorescent signal inside canalicular domains and CS3 cytosolic and canalicular surface signals were measured using ZEISS ZEN 2008 software and reported in arbitrary units.

### Immunoelectron Microscopy

For pre-embedding immunoelectron microscopy, cells were fixed, permeabilized, and labeled as described previously (Polishchuk et al., 2003). For cryo-immunoelectron microscopy, specimens were fixed, frozen, and cut using Leica EM FC7 ultramicrotome. Cryo sections were double labeled for LAMP1 and GFP. EM images were acquired using a FEI Tecnai-12 electron microscope. Morphometric analyses were performed using iTEM software (Olympus SIS).

### Statistical Analyses

Data are expressed as mean values ± SD. Statistical significance was computed using the Student’s two-tail t test. A p value < 0.05 was considered statistically significant. In all figures, ^∗^p < 0.05, ^∗∗^p < 0.01, and ^∗∗∗^p < 0.001.

### Additional Methods

Additional information on DNA constructs, adenoviruses, antibodies, immunoprecipitation, surface biotinylation, PLA, quantitative RT-PCR (qRT-PCR), RNAi, determination of β-Gal and β-Hex activities, atomic adsorption spectroscopy (AAS), and ICP-MS is provided in the Supplemental Experimental Procedures.

## Supplemental Information

Document S1. Supplemental Experimental Procedures and Figures S1–S5

Document S2. Article plus Supplemental Information
