# Supplementary material for: Wilson Disease Protein ATP7B Utilizes Lysosomal Exocytosis to Maintain Copper Homeostasis
Source: Dev Cell. 2014 Jun 23;29(6):686–700. doi: 10.1016/j.devcel.2014.04.033 (PMC4070386; doi:10.1016/j.devcel.2014.04.033)
Supplement: Document S1. Supplemental Experimental Procedures and Figures S1–S5 [file mmc1.pdf]

Developmental Cell, Volume *29*

Supplemental Information

Wilson Disease Protein ATP7B Utilizes Lysosomal Exocytosis to Maintain Copper Homeostasis

Elena V. Polishchuk, Mafalda Concilli, Simona Iacobacci, Giancarlo Chesi,
Nunzia Pastore, Pasquale Piccolo, Simona Paladino, Daniela Baldantoni,
Sven C.D. van Ijzendoorn, Jefferson Chan, Christopher J. Chang, Angela Amoresano,
Francesca Pane, Piero Pucci, Antonietta Tarallo, Giancarlo Parenti,
Nicola Brunetti-Pierri, Carmine Settembre, Andrea Ballabio, and Roman S. Polishchuk

Figure S1

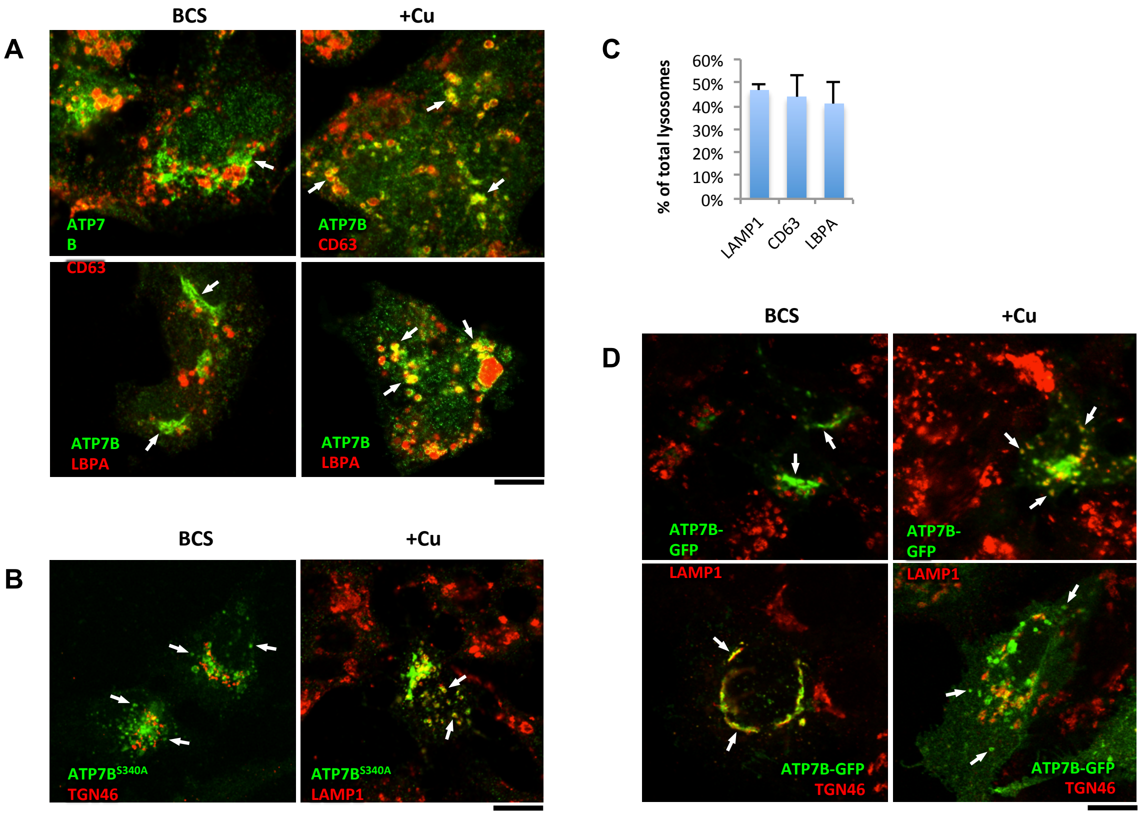

A
BCS
+Cu
ATP7
B
CD63
ATP7B
CD63
ATP7B
LBPA
ATP7B
LBPA
B
BCS
+Cu
ATP7B^{S340A}
TGN46
ATP7B^{S340A}
LAMP1
C
% of total lysosomes
60%
50%
40%
30%
20%
10%
0%
LAMP1
CD63
LBPA
D
BCS
+Cu
ATP7B-
GFP
LAMP1
ATP7B-
GFP
LAMP1
ATP7B-GFP
TGN46
ATP7B-GFP
TGN46

Figure S2

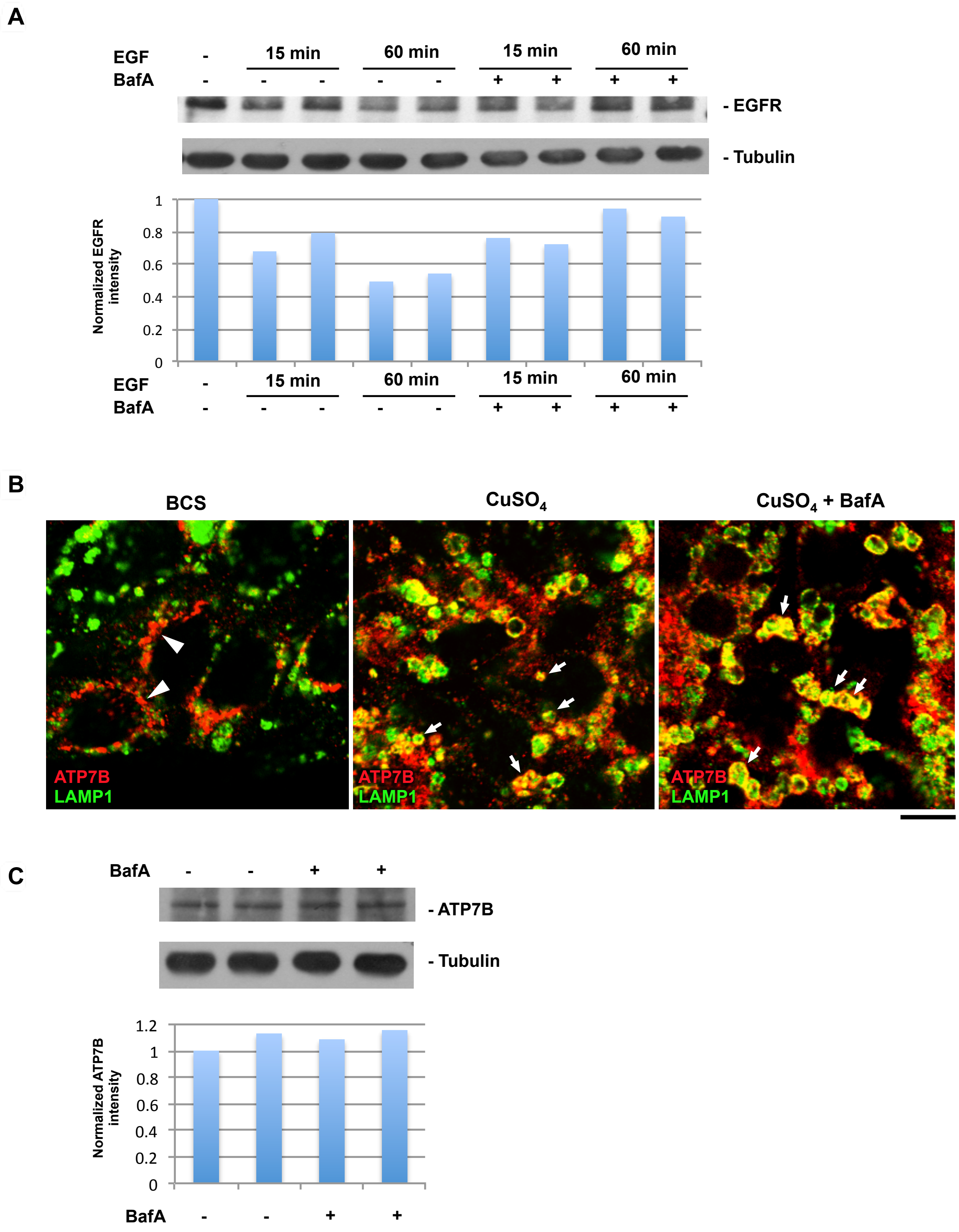
A
EGF
BafA
-
15 min
60 min
15 min
60 min
- EGFR
- Tubulin
Normalized EGFR intensity
1
0.8
0.6
0.4
0.2
0
B
BCS
$CuSO_4$
$CuSO_4$ + BafA
ATP7B
LAMP1
C
BafA
- ATP7B
- Tubulin
Normalized ATP7B intensity
1.2
1
0.8
0.6
0.4
0.2
0

Figure S3

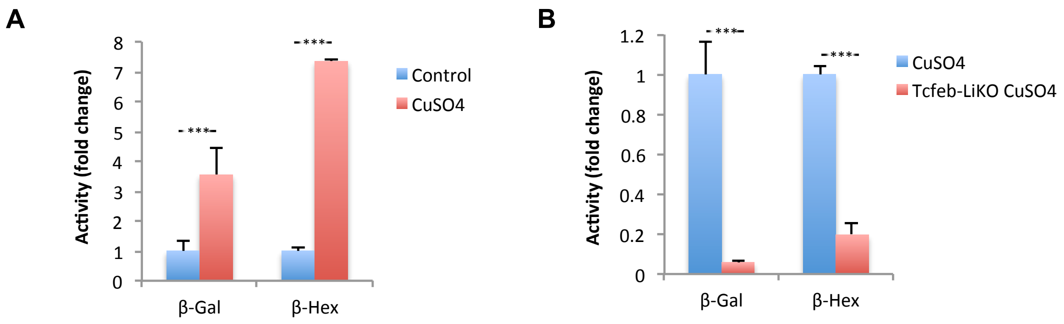

A
Activity (fold change)
8
7
6
5
4
3
2
1
0
***
***
β-Gal
β-Hex
Control
CuSO4
B
Activity (fold change)
1.2
1
0.8
0.6
0.4
0.2
0
***
***
β-Gal
β-Hex
CuSO4
Tcfeb-LiKO CuSO4

Figure S4

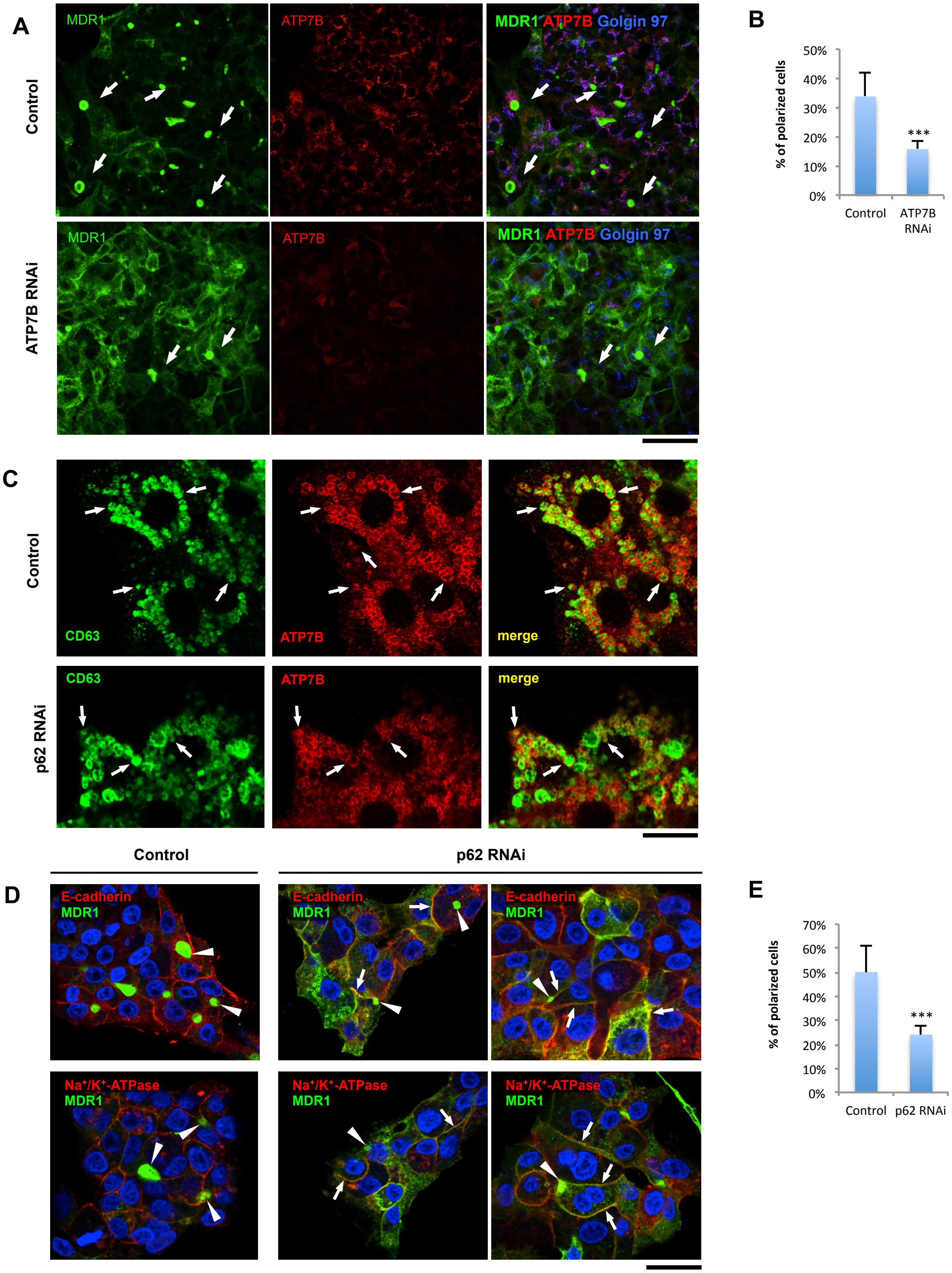
A
Control
ATP7B RNAi
MDR1
ATP7B
MDR1 ATP7B Golgin 97
B
% of polarized cells
50%
40%
30%
20%
10%
0%
***
Control
ATP7B RNAi
C
Control
p62 RNAi
CD63
ATP7B
merge
Control
p62 RNAi
D
E-cadherin
MDR1
Na+/K+-ATPase
E
% of polarized cells
70%
60%
50%
40%
30%
20%
10%
0%
***
Control
p62 RNAi

Figure S5

A

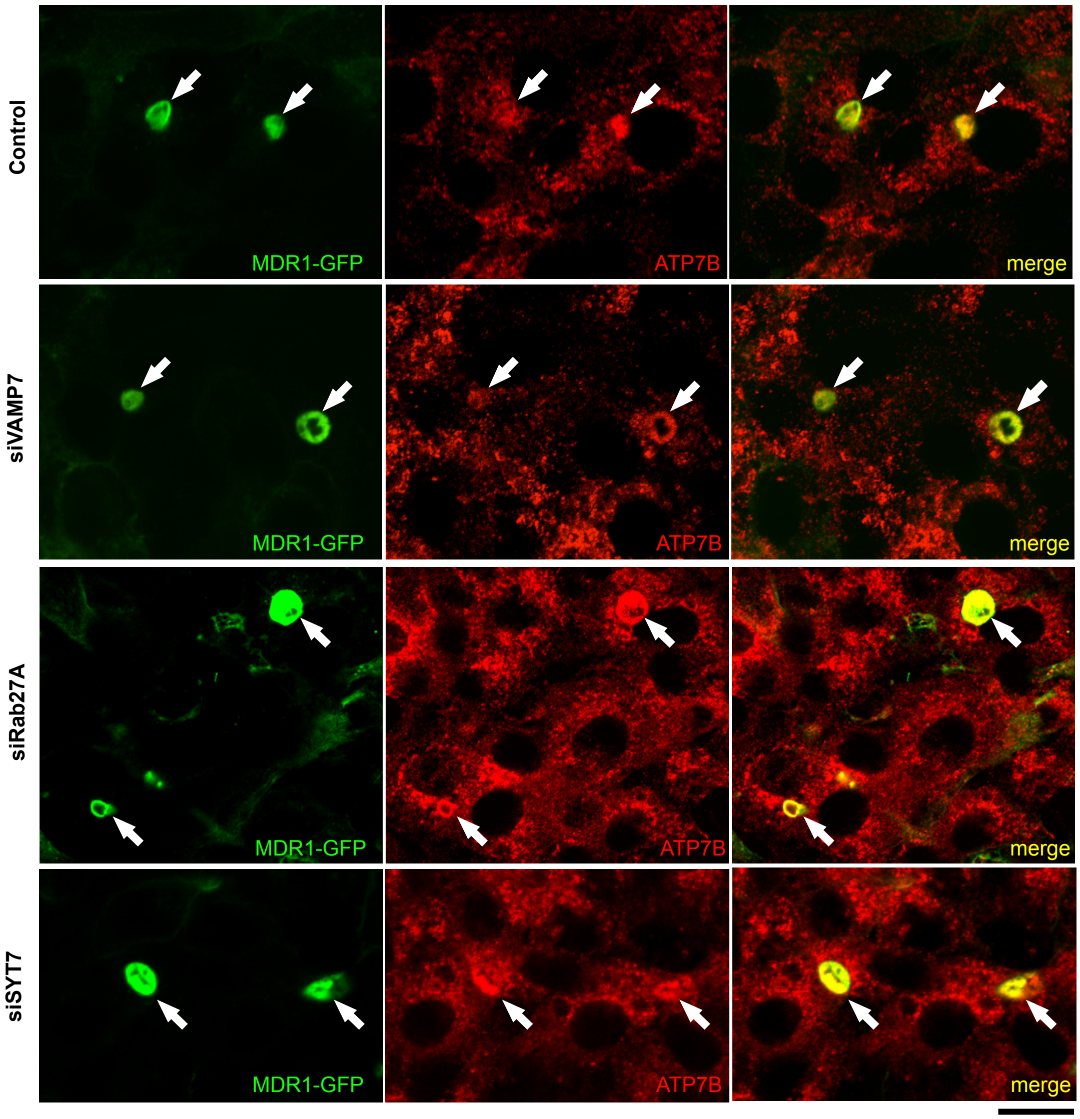
Control
siVAMP7
siRab27A
siSYT7
MDR1-GFP
ATP7B
merge

B

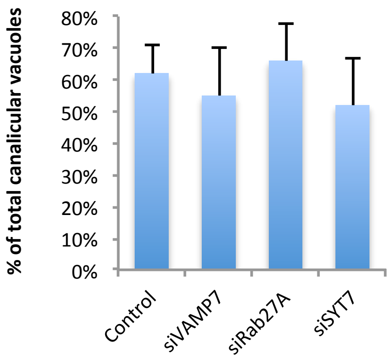
% of total canalicular vacuoles
80%
70%
60%
50%
40%
30%
20%
10%
0%
Control
siVAMP7
siRab27A
siSYT7

SUPPLEMENTAL FIGURE LEGENDS

Figure S1, related to Figure 1. Endogenous ATP7B and ATP7B-GFP traffic to lysosomal compartment in response to copper.

(A) HepG2 cells were fixed after overnight exposure to 200 µM BCS (left column) or after additional 2h incubation with 200 µM $CuSO_4$ (right column), stained for endogenous ATP7B and either CD63 (upper row) or LBPA (lower row) and investigated under confocal microscope. Arrows in the left row indicate ATP7B within the Golgi while arrows in the right row shows ATP7B within the structures containing lysosomal markers. **(B)** HepG2 cells expressing flag-tagged $ATP7B^{S340A}$ mutant were fixed after overnight exposure to 200 µM BCS and stained for endogenous either TGN46 or LAMP1. Arrows indicate $ATP7B^{S340A}$ in vesicular structures, which do not contain TGN46 but exhibit LAMP1 labeling. **(C)** Morphometric analysis shows the percentage (average ± SD, n=30 cells) of LAMP1-, CD63- or LBPA-positive structures that contain ATP7B from the total LAMP1-, CD63- or LBPA-positive structures in the cell. **(D)** HepG2 cells expressing ATP7B-GFP were fixed after overnight exposure to 200 µM BCS (left column) or after additional 2h incubation with 200 µM $CuSO_4$ (right column), stained for either LAMP1 (upper row) or TGN46 (lower row). Arrows in the left row indicate ATP7B-GFP within the Golgi while arrows in the right row shows ATP7B-GFP redistribution to the peripheral lysosome-like structures.

Scale bar: 4µm (A, B, D).

Figure S2, related to Figure 2. Delivery of ATP7B to lysosomes does not stimulate its degradation.

(A) In control experiment HeLa cells were starved overnight with Serum free DMEM, stimulated with 100 ng/ml EGF for 15 or 60 min in the presence or absence of 200 nM Bafilomycin A (BafA) and prepared for western blot to reveal EGF receptor (EGFR). Western blot shows EGFR levels to decrease 60 min after EGF stimulation, while 60 min incubation with BafA strongly inhibited the EGFR degradation. The graph exhibits normalized values of EGFR signal that correspond to each line in Western blot and shows that BafA prevents EGFR from degradation in lysosomes. **(B)** HepG2 cells were fixed

after overnight exposure to 200 μM BCS (left panel) or after additional 4h incubation with medium containing 200 μM $CuSO_4$ alone (central panel) or 200 μM $CuSO_4$ and 200 nM BafA (right panel), stained for endogenous ATP7B and LAMP1 and investigated under confocal microscope. ATP7B exhibits Cu-dependent redistribution from the Golgi (arrowheads) to LAMP1-positive structures (arrows) both in the absence and in the presence of BafA. This indicates that BafA does not inhibit Cu-dependent trafficking of ATP7B to the lysosomes. **(C)** HepG2 cells were treated for 4h with 200 μM $CuSO_4$ alone or 200 μM $CuSO_4$ and 200 nM BafA and then prepared for western blot to reveal endogenous ATP7B. Western blot does not show significant changes in the levels of endogenous ATP7B upon BafA treatment. The graph exhibits normalized values of ATP7B signal that correspond to each line in Western blot and indicates that BafA-mediated inhibition of lysosomal degradation does not change ATP7B levels although the protein traffics to the lysosomes (see panel B). Scale bar: 5.5μm (B).

Figure S3, related to Figure 5. TFEB suppression contrasts Cu-dependent exocytosis of lysosomal enzymes into the bile.

(A) Bile was collected from the control mice and animals that received $CuSO_4$ and the activity of β-Gal or β-Hex was measured in the bile (see methods). Normalized β-Gal or β-Hex activities (average ± SD, n=5 mice) in the bile increase upon Cu stimulation, indicating increase in lysosomal exocytosis at the canalicular surface of hepatocytes upon Cu stimulation. **(B)** Bile was collected from the control and Tcfeb-LiKO mice after stimulation with $CuSO_4$ and the activity of β-Gal or β-Hex in the bile was evalusted. Normalized β-Gal or β-Hex activities (average ± SD, n=3 mice) in the bile exhibit significant decrease in Tcfeb-LiKO mice indicating suppression of lysosomal exocytosis upon Tcfeb knockout.

Figure S4, related to Figure 7. Impact of ATP7B and p62 silencing on polarity and distribution of different markers in HepG2 cells.

(A) HepG2-MDR1 cells were grown to achieve maximal polarization, silenced for ATP7B, fixed at steady state conditions and stained for ATP7B and Golgin 97. Arrows indicate canalucular cysts, which decreased in number in ATP7B-silenced cells. **(B)**

Quantification reveals reduction in percentage of polarized cells (average ± SD; n=15 fields) forming apical cysts upon ATP7B silencing. **(C)** Control (upper row) or p62-silenced (lower row) HepG2 cells were treated first with BCS, then washed and exposed to $CuSO_4$. The cells were then fixed and stained for endogenous ATP7B and CD63. Arrows indicate ATP7B and CD63 within the same lysosome-like structures. **(D)** Control or p62-silenced HepG2-MDR1 cells were fixed and stained for either E-cadherin (upper row) or Na^+/K^+-ATPase. Arrowheads in all panels indicate MDR1 in canalicular cysts (which lack basolateral markers). Arrowheads indicate mistargeting of MDR1 to the basolateral surface in p62-silenced cells. **(E)** Quantification reveals reduction in percentage of polarized cells (average ± SD; n=15 fields) forming apical cysts upon p62 silencing. Scale bar: 13 μm (A), 4.2 μm (C), 8.2 μm (D).

Figure S5, related to Figure 7. Silencing of VAMP7, Rab27A and synaptotagmin 7 does not affect delivery of ATP7B to the canalicular surface of MDR1-GFP expressing HepG2 cells.

(A) Control or VAMP7, Rab27A and synaptotagmin 7 (SYT7)-silenced MDR1-GFP expressing HepG2 cells were treated first with BCS, then washed and exposed to $CuSO_4$ for 8 h. The cells were then fixed and stained for endogenous ATP7B. Arrows indicate ATP7B within MDR1 positive canalicular cysts. **(B)** The chart shows the percentage (mean ± SD, n=20 fields) of ATP7B-positive canalicular vacuoles. Scale bar: 5.5μm (A).

SUPPLEMENTAL EXPERIMENTAL PROCEDURES

Antibodies, plasmids and vectors.

The following antibodies were used. Rat anti-human ATP7B was kindly provided by Dr. S. Lutsenko), anti-human LAMP1 (H4A30) from Developmental Studies Hybridoma Bank, Iowa City, USA), rabbit anti-LAMP1 and mouse anti-α-tubulin, mouse anti-γ-adaptin, mouse anti-VSVG were from Sigma-Aldrich (St. Louis, USA), sheep anti-human TGN46 from AbD Serotec (Oxford, UK), rabbit anti-FAPP2, MPR giantin and rabbit anti-GFP for western blot analysis were kindly provided by Dr. A. De Matteis, TIGEM, Naples, Italy), rabbit anti-APPL1 and rabbit anti-EEA1 were from Cell Signaling (Danvers, MA), mouse anti-LBPA (6C4) antibody from Echelon (Salt Lake

City, USA), mouse anti-CD63 and mouse anti-p62 from Santa Cruz Biotechnology (California, USA), mouse anti-MRP2 from Enzo Life Sciences (Lausen, Switzerland), mouse anti-GM130, mouse anti-sorting nexin 1 and 2 (SNX1 and SNX2) from BD Transduction laboratories (California, USA), rabbit anti-human ATP7B for western blot analysis from Novus Biologicals (Littleton, USA), mouse anti-clathrin and rabbit anti-GFP antibody (ab290 for pre-embedding Immuno Electron Microscopy [IEM]) from Abcam (Cambridge, UK), mouse anti-golgin 97, mouse anti-transferrin receptor (TfR) and rabbit anti-GFP antibody (A-6455 for cryo IEM) secondary Alexa Fluor 488, 568, 633, 647 conjugated antibodies for immunofluorescence were from Invitrogen-Life Technologies (Grand Island, USA). Secondary peroxidase conjugate antibodies for western blot analysis were from Calbiochem (Darmstadt, Germany). GoldEnhance™ EM kit and 1.4nm gold-conjugated Fab' fragment of anti-rabbit IgGs were from Nanoprobes (Yaphank, NY 11980-9710, USA)

cDNA of ATP7B, GFP-tagged at the N-terminus, within pEGFP-C1 expression vector, was provided by Dominik Huster (Otto-vonGuericke-University, Magdeburg, Germany). cDNA of flag-tagged ATP7B^{S340A} mutant of ATP7B was from Svetlana Lutsenko (John Hopkins Medical School, Baltimore, MD). H1069Q mutation was introduced in ATP7B by site-directed mutagenesis using the QuickChange Kit (Stratagene, La Jolla, CA), using the following oligonucleotides: ATP7B-H1069Q-fw (5'-AGGCCAGCAGTGAACAACCCTTGGGCGTG-3') and ATP7B-H1069Q-rev (5'-CACGCCCAAGGGTTGTTCACTGCTGGCCT-3').

Cell culture, transfection and construction of recombinant adenoviruses

HepG2 cells were grown in Dulbecco's Modified Eagle's medium (DMEM) supplemented with 10% FCS (decomplemented at 56°C for 30 min), 2 mM L-glutamine, penicillin and streptomycin. For transfection of plasmids jetPEI TM-Hepatocyte DNA transfection reagent (Polyplus transfection TM, France) was used according to the manufacturer's instructions.

HepG2 cells stably expressing <u>m</u>ulti<u>d</u>rug <u>r</u>esistant protein 1(MDR1-GFP) were reported before (Slimane et al., 2003) and were grown as HepG2 cells but maintained with 0.8 mg/ml G418.

HeLa and HeLa CF7 cells were grown in (DMEM) supplemented with 10% FCS, 2 mM glutamine, penicillin and streptomycin. Cells were transfected with Trans IT LT1 Transfection reagent (Tema Ricerca SRL) according to the manufacturer instructions.

Generation of recombinant human type 5 adenovirus containing ATP7B-GFP or ATP7B^{H1069Q}-GFP, was performed by Vector biolabs (Philadelphia, PA, USA). To subclone the entire expression cassette, the following oligonucleotides were used: SV40revAscI (5'- AATGGCGCGCCTAAGATACATTGATGAGTTTGGA -3') and CMVfwAscI (5'- TCCGGCGCGCCTGTTATTAATAGTAATCAATTACGG -3'). Cells were infected with first generation adenovirus containing ATP7B-GFP or ATP7B^{H1069Q}-GFP respectively with a Multiplicity Of Infection (MOI) of 50 and 200 virus particles per cell respectively. Helper dependent adenovirus containing TFEB was reported previously (Pastore et al., 2013) and utilized at MOI 1000 particles per cell to infect polarized HepG2 cells.

Trafficking assay and copper treatment.

To investigate localization of ATP7B at the different Cu load cells were treated with 200 μM Cu-chelating agent BCS and with different concentrations of $CuSO_4$ (20, 40, 100, 200 and 500 μM) in culture medium at 37°C for 2 or 8 h. To compare trafficking of ATP7B with a conventional cargo protein (VSVG) cells were first incubated with BCS (overnight), then infected with VSV as described previously (Polishchuk et al., 2003), incubated at 20°C in the presence of BCS (to accumulate both proteins in the Golgi) and finally warmed to 32°C and treated with $CuSO_4$ (to activate post-Golgi transport of both proteins) in the presence of 100 μg/ml cycloheximide. Tannic acid at the 0.5% concentration was added in transport experiments during release of 20°C block to inhibit fusion of post-Golgi transport carriers with the PM (Polishchuk et al., 2004).

RNA interference.

Small interfering RNA (siRNA) oligonucleotides targeting the ATP7B, mucolipin1, p62 (DNCT4), Vamp7, SYT7, Rab27A were purchased from Sigma-Aldrich. Following siRNA were utilized:

siATP7B-1 CCAAUUGAUAUUGAGCGGUUA

siATP7B-2 GAUAUUGAGCGGUUACAAA

siDNCT4(p62)-1 GCUCUAUCCUCGCCACAAA

siDNCT4(p62)-2 GCUUCAAGAUGAAGCAUGA

siMucolipin1 CCCACAUCCAGGAGUGUAA

siVamp7-1 CACAUACUGACAGAUGGUA

siVamp7-2 CUGAGAAUAAGGGCCUAGA

siSYT7-1 GAGUCCUUCGCCUUCGAUA

siSYT7-2 GAAUGUCGAGGAUAGUAUA

siRab27A-1 GAUGCAUGCAUAUUGUGAA

siRab27A-1 CAUUAGACCUACGAAUAAA

Scrambled siRNAs were used as a control. HepG2 cells were transfected with siRNA using Darmafect4 (Dharmacon, Pittsburgh, USA), according to manufacturer instructions.

RNA Preparation and Q-PCR

Total RNAs from control cells and cells silenced for ATP7B or mucolipin1 as well as from the cells infected with HDAd-TFEB were purified by QIAshredder (Qiagen) and extracted with RNeasy Protect Mini Kit (Qiagen) using standard conditions. Total RNA (1 μg) was reverse-transcribed by QuantiTect Reverse Transcription kit (Qiagen) according to the manufacturer's instructions. Q-PCR experiments were performed using Light Cycler 480 Syber Green MasterMix (Roche) for cDNA amplification and in LightCycler 480 II (Roche) for signal detection. Q-PCR results were analyzed using the comparative Ct method normalized against housekeeping gene β-Actin.

The specific primer pair:

β-ACTIN forward (5'-AAGAGCTACGAGCTGCCTGA-3')

β-ACTIN reverse (5'-GACTCCATGCCCAGGAAGG-3')

ATP7B forward (5'- TCTCTGGTCATCCTGGTGGTT-3')

ATP7B reverse (5'- GGGCTTCTGAGGTTTTGCTCT-3')

Mucolipin1 forward (5'- GGCCAACGACACATTTGAC -3')

Mucolipin1 reverse (5'- TTTCCAAGAGGGTGAGATCG -3')

hTFEB forward (5'-CCAGAAGCGAGAGCTCACAGA-3')

hTFEB reverse (5'-TGTGATTGTCTTTCTTCTGCCG-3')
p62 (DCTN4) forward (5'-TGAGAACCTCACCCATGTGAC-3')
p62 (DCTN4) reverse (5'-ATCCTTGCCAGCTAAAACGAG-3')

Mice and treatment

Conditional *Tcfeb*-flox mouse line generation was described previously (Settembre et al., 2013). Mice were maintained in a C57BL/6 strain background. 2 months-old males mice were used. As control animals we utilized TcFEB loxP/loxP mice that did not carry the Albumin Cre transgene. To express ATP7B-GFP in liver both control and *Tcfeb*-flox mice were subjected to retro-orbital intravenous injection with Adeno-ATP7B-GFP ($5x10^{10}$ particles per mouse) 3 days before the experiment. To stimulate Cu excretion from liver both control and *Tcfeb*-flox mice received 0.125% $CuSO_4$ in water 4h before the animals were sacrificed (Gross et al., 1989). Liver tissue was rapidly dissected from the mice, fixed and processed for electron microscopy. All experiments were approved by the Committee on Animal Care at Baylor College of Medicine and conform to the legal mandates and federal guidelines for the care and maintenance of laboratory animals.

Immunofluorescence.

Cells were fixed for 10 min with 4% paraformaldehyde (PFA) in 0.2 M HEPES followed by incubation with blocking/permeabilizing solution: 0.5% bovine serum albumin (BSA), 0.1% saponin, 50 mM NH_4Cl in PBS for 20-30 min. Primary and secondary antibodies were diluted in blocking/permeabilizing solution and added to the cells for 1h/overnight or 45 min respectively. Samples were examined with a ZEISS LSM 700 or LSM 710 confocal microscopes equipped with a 63X 1.4 NA oil objective.

For fluorescent Cu detection with CS3 cells were incubated with 5 µM CS3 solution for 15 min at 37°C. CS3 was excited with 561 nm laser of LSM710 and its emission was collected from 565 to 650 nm. Co-localization module of ZEISS ZEN 2008 software was used to measure co-localization of ATP7B with different intracellular markers. ATP7B fluorescent signal inside canalicular domains and CS3 cytosolic and canalicular surface signals were measured using ZEISS ZEN 2008 software and reported in arbitrary units (au).

Proximity ligation assay (PLA)

In-situ ATP7B-GFP and P62 interaction, revealed as red fluorescent dots, was detected using the Duolink II PLAkit (Olink Bioscience, Uppsala, Sweden), according to the manufacturer's instructions. As a positive control, cells were co-transfected with $ATP7B^{H1069Q}$-GFP and its validated interactor 3XFLAG-CRYAB (D'Agostino et al., 2013) and labelled with an anti-GFP antibody in combination with an anti-Flag antibody. As a negative control, cells were transfected with ATP7B-GFP and labelled with an anti-GFP antibody in combination with the antibody against the luminal domain of transferrin receptor (TfR) (Invitrogen) to demonstrate no interaction between ATP7B-GFP and TfR (D'Agostino et al., 2013). Coverslips were stained with Hoechst, mounted in Mowiol and analyzed by confocal immunofluorescence microscopy.

Immuno-electron microscopy.

For pre-embedding immuno-electron cells microscopy were fixed, permeabilized and labeled as described previously (Polishchuk et al., 2003). Briefly, the cells were fixed with mixture of 4%PFA and 0.05% glutaraldehyde in 0.2 M HEPES for 15 min and with 4%PFA alone for 30 min, followed by incubation with blocking/permeabilizing solution: 0.5% bovine serum albumin (BSA), 0.1% saponin, 50 mM NH_4Cl in PBS for 20-30 min. Primary anti-GFP antibody and 1.4nm gold-conjugated Fab' fragment of anti-rabbit IgGs were diluted in blocking/permeabilizing solution and added to the cells overnight or for 2h respectively. GoldEnhance™ EM kit was used to enhance ultrasmall gold particles. Then cells were scraped, pelleted, post-fixed in OsO_4 and uranyl acetate and embedded in Epon. For cryo immuno-electron microscopy HepG2 cells or small 1 mm^3 pieces of liver tissue from sacrificed mice were rapidly washed in PBS 1X and fixed immediately with a mixture of 2% freshly prepared paraformaldehyde and 0.2% glutaraldehyde in 0.1 M phosphate buffer for 2 h at room temperature. Before freezing in liquid nitrogen cell and tissue gelatin blocks were immersed in 2.3 M sucrose. From each sample, thin plastic or cryo sections were cut using Leica EM UC7 or Leica EM FC7 ultramicrotomes respectively (Leica Mycrosystems, Vienna, Austria). Cryo sections were double labeled with antibodies against LAMP1 and GFP. EM images were acquired from thin sections

using a FEI Tecnai-12 electron microscope (FEI, Eindhoven, Netherlands) equipped with a VELETTA CCD digital camera (Soft Imaging Systems GmbH, Munster, Germany). Morphometric analysis of lysosomal size, distance between lysosomes and PM, distribution of ATP7B among different intacellular compartments was performed using iTEM software (Olympus SYS, Germany).

Cell surface biotinylation and immunoprecipitation.

HeLa and Hela CF7 cells were infected with adenovirus carrying either ATP7B-GFP or ATP7B^{H1069Q}-GFP. The day after infection cells were rinsed twice with phosphate-buffered saline (PBS) containing 0.1 mM $CaCl_2$ and 1 mM $MgCl_2$ followed by two successive 20 min incubations at 4°C with 0.5 mg/ml EZ-Link Sulfo-NHS-Biotin (Pierce) diluted in PBS. The biotinylation reaction was stopped by washing the cells with PBS/NH_4Cl quenching solution for 10 min at 4°C. Cells were then solubilized at 4°C in a lysis buffer containing 0.5 % Triton X-100, 20 mM Tris/HCl (pH 7.4), 150 mM NaCl, 1mM EDTA (pH: 8), 0.5% Np-40, 10% Glycerol, supplemented with 1× protease inhibitor cocktail (Sigma). The lysate was incubated with streptavidin beads (Pierce) at 4°C overnight. After incubation, beads were pelleted by centrifugation at 4000 rpm for 1 min. The beads were then washed in lysis buffer (20 mM Hepes pH 7.4, 150 mM NaCl, 10% Glycerol, 0.1% Triton X-100), supplemented with 1× protease inhibitor cocktail. Biotinylated proteins were eluted with SDS sample buffer, containing 100 mM β-mercaptoethanol and analyzed by immunoblot analysis. These cells were solubilized in lysis buffer for 10 minutes at room temperature (RT). The mixture was placed into a microfuge tube, kept on ice for 10 min, and then spun at 14 000 rpm for 15 min at 4°C. Cell lysates of biotinylated samples or total lysates of HepG2 cells were subjected to SDS-PAGE and probed for GFP and ATP7B respectively. Western blot analysis was also performed in HepG2 cells infected with adenovirus carrying TFEB or treated with ATP7B- or p62-specific siRNAs.

Immunoprecipitation to test Cu-specific interactions between ATP7B and p62 was as follows. Cell lysates from polarized HepG2 cells were incubated with anti-p62 antibody. Then protein G sepharose beads (Sigma) were added to each specimen and immune complexes were collected by centrifugation. The beads were then washed and

immunoprecipitated proteins were eluted, separated by SDS-PAGE and analyzed by Western blot.

Copper detection by atomic absorption spectroscopy (AAS) or inductively coupled plasma mass spectrometry (ICP-MS).

To determine intracellular Cu concentrations, we first collected the cell pellets into a mixture of 20mM Na_3PO_4 and 10mM NaCl, and lysed through sonication (Sonics Vibra-cells). The protein concentration in each sample was evaluated using Bradford Protein Assay (BioRad, Segrate, Italy). To evaluate Cu concentrations in biliary cysts, polarized HepG2 cells were exposed to 2mM EDTA in PBS for 5 min. This treatment breaks tight junctions and allows content of biliary cyst to be released into extracellular space (Zegers and Hoekstra, 1997). Then extracellular fluid was collected and processed for spectroscopy. Cu concentrations in cell lysates were measured using an atomic absorption spectrometer (AAnalyst 100, PerkinElmer, Wellesley, MA, USA), equipped with a graphite atomizer apparatus and autosampler (AS 800, PerkinElmer, Wellesley, MA, USA). Cu concentration in the cell lysates or in the material released from apical cysts was analyzed by ICP-MS. An aliquot of each sample was transferred into polystyrene liners, diluted 1:10 v/v with 5% HNO_3 and finally analyzed with an Agilent 7700 ICP-MS (Agilent Technologies, Santa Clara, CA, USA), equipped with a frequency-matching RF generator and 3rdgeneration Octopole Reaction System (ORS^3), operating with helium gas in ORF. The following parameters were used: radiofrequency power 1550 W, plasma gas flow 14 L/min; carrier gas flow 0.99 L/min; He gas flow 4.3 mL/min. ^{103}Rh was used as an internal standard (50 μg/L final concentration). Multi-element calibration standards were prepared in 5% HNO_3 at 4 different concentrations (1, 10, 50, and 100 μg/L). All values of Cu concentration were normalized for protein content in corresponding cell lysates.

Determination of β-galactosidase (β -GAL) and β-hexosaminidase (β-Hex) activities.

Activities of lysosomal enzymes of β-Gal or β-Hex were analyzed in mice liver homogenates and in bile collected from the gall bladder using 4-methylumbellipheryl-β-D-galactopyranoside (Sigma) as a substrate for β-Gal and 4-Methylumbelliferyl N-

acetyl-β-D-glucosaminide (Sigma) as a substrate for β-Hex. β-Gal activity was also measured in the biliary cysts of polarized HepG2-MDR1 cells. To this end the cells were exposed to 2mM EDTA in PBS for 5 min to open the tight junctions and, thus, to collect the content of the apical vacuoles. Corresponding cell lysates were also processed for the enzyme activity analysis. Fluorescence of samples was measured using a Fluoroskan Ascent FL spectrofluorometer (Thermo Electron Corporation) at the exitation and emission wavelengths of 355 and 460 nm, respectively. The results were expressed in nmol methylumbellipheryl (MUP, M1381 Sigma) released per μl of the specimen over 1 hour and normalized for protein concentration in corresponding liver lysate (bile and liver tissue specimens) or cell lysate (canalicular cyst content and HepG2-MDR1 cells specimens). Then the activities of enzymes in bile were normalized for those in liver, while the activities of enzymes in canalicular cysts were normalized for those cell lysates. In each experiment the fold change of activity in either bile or canalicular cyst content was quantified.

Statistical analyses

Data are expressed as mean values ± standard deviation. Statistical significance was computed using the Student's 2 tail t-test. A p-value <0.05 was considered statistically significant. In all figures * means p-value <0.05, ** p-value <0.01, *** p-value <0.001.

SUPPLEMENTAL REFERENCES

D'Agostino, M., Lemma, V., Chesi, G., Stornaiuolo, M., Cannata Serio, M., D'Ambrosio, C., Scaloni, A., Polishchuk, R., and Bonatti, S. (2013). The cytosolic chaperone alpha-crystallin B rescues folding and compartmentalization of misfolded multispan transmembrane proteins. J Cell Sci *126*, 4160-4172.

Gross, J.B., Jr., Myers, B.M., Kost, L.J., Kuntz, S.M., and LaRusso, N.F. (1989). Biliary copper excretion by hepatocyte lysosomes in the rat. Major excretory pathway in experimental copper overload. J Clin Invest *83*, 30-39.

Pastore, N., Blomenkamp, K., Annunziata, F., Piccolo, P., Mithbaokar, P., Maria Sepe, R., Vetrini, F., Palmer, D., Ng, P., Polishchuk, E., *et al.* (2013). Gene transfer of

master autophagy regulator TFEB results in clearance of toxic protein and correction of hepatic disease in alpha-1-anti-trypsin deficiency. EMBO Mol Med *5*, 397-412.

Polishchuk, E.V., Di Pentima, A., Luini, A., and Polishchuk, R.S. (2003). Mechanism of constitutive export from the golgi: bulk flow via the formation, protrusion, and en bloc cleavage of large trans-golgi network tubular domains. Mol Biol Cell *14*, 4470-4485.

Polishchuk, R., Di Pentima, A., and Lippincott-Schwartz, J. (2004). Delivery of raft-associated, GPI-anchored proteins to the apical surface of polarized MDCK cells by a transcytotic pathway. Nat Cell Biol *6*, 297-307.

Settembre, C., De Cegli, R., Mansueto, G., Saha, P.K., Vetrini, F., Visvikis, O., Huynh, T., Carissimo, A., Palmer, D., Klisch, T.J., *et al.* (2013). TFEB controls cellular lipid metabolism through a starvation-induced autoregulatory loop. Nat Cell Biol *15*, 647-658.

Slimane, T.A., Trugnan, G., Van, I.S.C., and Hoekstra, D. (2003). Raft-mediated trafficking of apical resident proteins occurs in both direct and transcytotic pathways in polarized hepatic cells: role of distinct lipid microdomains. Mol Biol Cell *14*, 611-624.

Zegers, M.M., and Hoekstra, D. (1997). Sphingolipid transport to the apical plasma membrane domain in human hepatoma cells is controlled by PKC and PKA activity: a correlation with cell polarity in HepG2 cells. Journal Cell Biol *138*, 307-321.
